# Spatial Multiomics Reveals Intratumoral Immune Heterogeneity with Distinct Cytokine Networks in Lung Cancer Brain Metastases

**DOI:** 10.1158/2767-9764.CRC-24-0201

**Published:** 2024-11-06

**Authors:** Gustav Christensson, Matteo Bocci, Julhash U. Kazi, Geoffroy Durand, Gustav Lanzing, Kristian Pietras, Hugo Gonzalez Velozo, Catharina Hagerling

**Affiliations:** 1 Department of Experimental Medical Science, Lund University, Lund, Sweden.; 2 Lund University Cancer Centre (LUCC), Lund University, Lund, Sweden.; 3 Division of Translational Cancer Research, Department of Laboratory Medicine, Lund University, Lund, Sweden.; 4 Division of Clinical Genetics, Department of Laboratory Medicine, Lund University, Lund, Sweden.; 5 Department of Anatomy, University of California, San Francisco, San Francisco, California.; 6 Laboratory of Tumor Microenvironment and Metastasis, Centro Ciencia & Vida, Santiago, Chile.

## Abstract

The tumor microenvironment of brain metastases has become a focus in the development of immunotherapeutic drugs. However, countless patients with brain metastasis have not experienced clinical benefit. Thus, understanding the immune cell composition within brain metastases and how immune cells interact with each other and other microenvironmental cell types may be critical for optimizing immunotherapy. We applied spatial whole-transcriptomic profiling with extensive multiregional sampling (19–30 regions per sample) and multiplex IHC on formalin-fixed, paraffin-embedded lung cancer brain metastasis samples. We performed deconvolution of gene expression data to infer the abundances of immune cell populations and inferred spatial relationships from the multiplex IHC data. We also described cytokine networks between immune and tumor cells and used a protein language model to predict drug–target interactions. Finally, we performed deconvolution of bulk RNA data to assess the prognostic significance of immune–metastatic tumor cellular networks. We show that immune cell infiltration has a negative prognostic role in lung cancer brain metastases. Our in-depth multiomics analyses further reveal recurring intratumoral immune heterogeneity and the segregation of myeloid and lymphoid cells into distinct compartments that may be influenced by distinct cytokine networks. By using computational modeling, we identify drugs that may target genes expressed in both tumor core and regions bordering immune infiltrates. Finally, we illustrate the potential negative prognostic role of our immune–metastatic tumor cell networks. Our findings advocate for a paradigm shift from focusing on individual genes or cell types toward targeting networks of immune and tumor cells.

**Significance:**

Immune cell signatures are conserved across lung cancer brain metastases, and immune–metastatic tumor cell networks have a prognostic effect, implying that targeting cytokine networks between immune and metastatic tumor cells may generate more precise immunotherapeutic approaches.

## Introduction

Brain metastases affect up to 20% of patients with cancer (1, 2), with lung cancer being the most common cancer type to colonize the brain (3, 4). A brain metastasis diagnosis represents the most devastating complication of advanced cancer, with very few effective treatments. The brain metastasis tumor microenvironment has gained attention in the emergence of immunotherapeutic drugs (5). Importantly, the brain differs greatly from other metastatic sites, in part due to its special anatomical structures but also due to its unique tissue-resident cells (6). Identifying new therapeutic targets within the tumor microenvironment of brain metastases holds the promise for enhancing treatment efficiency.

Recent breakthroughs in immunotherapy have started to transform the management of lung cancer brain metastases (5), as evidenced by several ongoing clinical trials (7). The immune checkpoint inhibitor pembrolizumab demonstrated an intracranial response rate of 33% in non–small cell lung cancer (NSCLC) with PD-L1 expression above 1% (8), and a combination of the immunotherapeutic agents nivolumab and ipilimumab proven efficient in targeting melanoma brain metastases (9) has also shown promise for lung cancer brain metastases (10, 11). However, there is a lack of response in a significant proportion of patients with brain metastases. Thus, an understanding of the immune cell composition and tumor–immune cellular networks within brain metastases may help to generate more precise immunotherapeutic approaches and stratify patients into different risk groups.

Although new studies are beginning to spatially unravel the immune landscape in lung cancer brain metastases (12, 13), most have only examined a limited number of regions per tissue sample, without exploring intratumor heterogeneity. In this study, we provide a detailed spatial understanding of the immune landscape in lung cancer brain metastases by performing spatial multiomics [whole transcriptomic atlas (WTA) profiling with extensive multiregional sampling and multiplex imaging]. We investigate how immune and metastatic tumor cells (MTC) communicate and shape the tumor microenvironment and evaluate the prognostic and therapeutic potential of immune–MTC cellular networks. Our emphasis on multiregional sampling and multiomics enhances existing knowledge on spatial heterogeneity and multicellular networks as potential immunotherapeutic targets.

## Materials and Methods

### Ethics

Ethical approval was obtained from the regional committee in Lund, Sweden (Dnr 2019-04998). Informed written consent was obtained by the patients prior to participation in the study, which was conducted in agreement with the Declaration of Helsinki.

### Patient cohort

The NSCLC brain metastasis cohort analyzed included 27 patients diagnosed at Skåne University Hospital, Sweden, between 2015 and 2020 (Supplementary Table S1). The median age at brain metastasis diagnosis was 66 years, ranging from 52 to 75 years of age. Information about age was missing for one patient. The median follow-up for patients alive at the last follow-up was 16 months from the brain metastasis diagnosis and 29 months from the initial NSCLC diagnosis.

Formalin-fixed and paraffin-embedded (FFPE) lung cancer brain metastases from all patients were obtained from the Department of Pathology at the Skåne University Hospital, Sweden. Two to four representative 1-mm-wide tumor cores were selected per patient and subsequently assembled in a tissue microarray (TMA).

### Immunohistochemistry

Immunohistochemistry (IHC) was performed at the Department of Pathology at the Skåne University Hospital, Sweden. A section (4 μm) of the TMA was stained with monoclonal mouse anti-human CD45 antibody (clone 2B11 + PD7/26, DAKO, RRID: AB_2890927) with an automated protocol on the BenchMark ULTRA IHC/ISH System (Roche Diagnostics). The percentage of CD45^+^ cells was annotated by a pathology resident. The cohort was stratified into CD45^low^ and CD45^intermediate/high^ groups using the 25th percentile (3.50%), inclusive, as a cutoff for including patients in the CD45^intermediate/high^ group. Differences in survival between the two groups was calculated using the log-rank test for equal survival distributions using the “survdiff” function from the R package “survival.”

### Spatial transcriptomics

Four patients from the cohort, representative of the intermediate/high immune infiltration group, were further selected for spatial multiomics. Their brain metastasis FFPE blocks were sectioned at a thickness of 5 μm before being placed onto glass slides. The FFPE slides were incubated with ISH oligonucleotide probes from the Human NGS WTA RNA 1.0 Library overnight by the Center of Translational Genomics at Lund University according to the manufacturer’s protocol. The slides were stained for DNA (SYTO13 dye, 500 nmol·dm^−3^, NanoString), pan-cytokeratin (AE1/AE3 clone, conjugated to Alexa Fluor 532, 1.5 mg·dm^−3^, NanoString, RRID: AB_2924722), and CD45 (anti-CD45 antibody, PD7/26 + 2B11, conjugated to Alexa Fluor 647, 10 mg·dm^−3^, Novus, RRID: AB_3287666) and loaded into NanoString GeoMx Digital Spatial Profiler (NanoString), which scanned the slides to yield one high-resolution fluorescent image per slide.

The complementary NanoString GeoMx software was used to draw regions of interest (ROI), which comprised the tumor core (TC), tumor–immune (TI) interface, and I ROIs. TI interfaces were arbitrarily defined as including up to two rows of MTCs bordering immune infiltrates. The mean area for the I, TI, and TC ROIs were 26,899, 39,935, and 35,262 square micrometers, respectively, with a mean of 29, 37, and 30 nuclei per ROI type. On average, each TC ROI was 192 μm from the nearest I ROI on the two-dimensional space of the slide, with a range of 33 to 561 μm (Supplementary Fig. S2C; Supplementary Table S2). UV light–cleaved probes were sequenced and counted using the NextSeq2000 (Illumina).

### Spatial transcriptomics data analysis

The GeoMx data were imported into R Studio (R Studio, PBC), running R version 4.3, and analyzed using the GeomxTools package (14). We filtered out ROIs with fewer than 1,000 mRNA reads to ensure adequate amounts of data. Additionally, only ROIs with at least 80% of reads trimmed, stitched, and aligned were kept. To further enhance data reliability, the minimum sequencing saturation was mandated to 50%, the minimum negative control count was set to two, and the number of counts in the No Template Control (NTC) well was capped to 1,000. Lastly, the minimum number of nuclei per ROI was set to 40, whereas the minimum ROI area was set to 12,000 square micrometers. Taken together, 101 ROIs passed these stringent cutoffs and were included. The limit of quantification for each ROI was calculated based on the distribution of negative control probes to estimate the lower limit of reasonable gene expression per segment. One ROI with a gene detection rate of less than 5% (i.e., with less than one in 20 genes detected above that ROI’s limit of quantification) was also excluded—so were four ROIs belonging to neither TC, TI interface, nor immune-infiltrating regions—bringing the final number of ROIs to 96. Genes detected in less than or equal to 5% of ROIs were also discarded, bringing the total number of features from 18,677 to 9,390, including negative probes.

In the subsequent phase of our analysis, we normalized our dataset using the Q3 normalization method instead of normalizing to the negative probe counts to prevent any unstable effects of the background reads. This was achieved using the “normalize” function from the NanoStringNCTools package.

We visualized the dimensionality of our ROI dataset using Uniform Manifold Approximation and Projection (UMAP), with coordinates calculated using the logarithm in base two of the Q3-normalized gene expression data for all genes. The UMAP settings included 15 nearest neighbors, two components, and Euclidean distance calculation between data points. Additionally, we clustered the dataset using the *k*-means algorithm, using the gap statistic method to guide us in setting the number of clusters. We then analyzed the I ROIs using the SpatialDecon package (15, 16). We downloaded single‐cell RNA sequencing (scRNA-seq) expression data from a public dataset which had studied primary and metastatic lung cancers and described 8 immune cell populations and 49 subpopulations of immune cells in lung cancer central nervous system metastases (17). We used these data to create a cell profile matrix using the provided function (“create_profile_matrix”), including cell types with a prevalence of at least 0.50% (145 cells) in the reference dataset (29,060 cells). We removed malignant profiles and used this matrix to run the “spatialdecon” function on the I ROIs, creating four MTC profiles to regress out the inferred tumor expression from our ROIs. The ROIs were clustered by their estimated cell abundances (termed “beta” values) using hierarchical clustering, and a bar plot was used to visualize the cell compositions. Based on this, the I ROIs could be divided into five clusters, which were plotted on UMAP plots.

ROIs containing MTCs from the TI interface were viewed in QuPath 0.4.0 (18) and annotated with the identity of the adjacent I ROI. TI ROIs adjacent to I ROIs with different signatures were annotated with both identities and excluded from the analysis. TI ROIs without an adjacent I ROI were labeled as “Unknown.” The TI ROIs were also visualized on UMAP plots. Differential gene expression analysis was once again used to compare each cluster of immune signature–associated TI ROIs with remaining TI ROIs, and the results were analyzed using gene set enrichment analysis (GSEA) with the “gseGO” function in clusterProfiler (19–21). For this analysis, the genes were ordered by the product of the log_2_–fold change (log_2_FC) and the negative logarithm in base 10 of the *P* value. The “minGSSize” parameter was set to 10, whereas the “maxGSSize” parameter was set to 500. TI ROIs marked as “unknown” or TI ROIs between two different immune signatures were excluded from this analysis. For this test, the genes were ranked in descending order by the product of their log_2_FC and the negative logarithm in base 10 of the *P* value. The differential expression comparison was repeated by only considering genes coding for cytokines using the list of cytokines retrieved from the “Secreted signaling” database in the CellChat R package (RRID: SCR_021946; refs. 22, 23). Finally, all of the above deconvolution and downstream analyses were repeated using a second scRNA-seq study, which had also profiled brain metastases (24). In the latter case, only the datasets sequenced from lung cancer brain metastases were downloaded.

We analyzed the TI ROIs and I ROIs using the CellChat package (22). Although the latest version of this package can handle spatial data (23), we chose to ignore the spatial component of our ROIs as the package is not formally designed to analyze GeoMx data. We log-transformed the normalized gene expression data and selected a ligand–receptor interaction database containing a subset focused on secreted signaling interactions (“Secreted signaling”). The TI ROIs were grouped by their adjacent I ROI according to the spatial deconvolution result. The communication probability was computed using the “computeCommunProb” function with a 5% truncated mean to calculate the average gene expression in each ROI. This meant that the average gene expression was calculated to zero if the percent of cells expressing that gene was less than 5%. Interactions were computed even amongst ROIs that were not adjacent to each other. We explored the probabilities and *P* values associated with each interaction and summarized the number of ligand–receptor interactions between each group of ROIs.

We next filtered the dataset to include only TI and TC ROIs and performed differential expression (DE) analysis to find differentially expressed genes between TC and TI ROIs for all patients. We conducted a GSEA using the “gseGO” function from clusterProfiler (19–21), with similar methodology as described above, to identify enriched biological processes associated with differentially expressed genes. The analysis was also repeated per patient. Next, we used the SpatialOmicsOverlay package to visualize the gene expression of selected genes on the slide micrograph, as well as a selection of metadata for the I ROIs (25).

The distances from the center of each TI and TC ROI centroid to the nearest immune infiltrate were measured on the slide micrographs using QuPath. For TI ROIs, the distance was forced to be zero. Summary statistics, including mean, median, and quantiles, were calculated to gain insights into the distribution characteristics. The tumor ROIs for each patient were plotted in UMAP space, and the distribution of the ROIs was observed to see if it correlated with the distance from the immune infiltrates.

Finally, we compiled a list of all DE genes for TC and TI ROIs in patient 19 with an absolute log_2_FC value more than 0.50 and a *q*-value less than 0.10, the latter calculated across all patients’ comparisons. We used a pretrained protein language model coupled with contrastive learning to gather annotated molecules from the DrugBank database, in which the molecules were methodically paired with proteins encoded by the genes in either the TC or the TI gene list. The gene set from the TC comprised 759 genes, whereas the TI gene set included 558 genes. We reported protein–molecule interaction for FDA-approved molecules and genes which passed a ConPLex threshold of 0.92 based on recommendations (26).

### Bulk RNA-seq and deconvolution

RNA extraction was performed on all 27 FFPE samples of lung cancer brain metastases included in the cohort. Two or three 10-μm sections from freshly cut FFPE tissue were deparaffinized with xylene. RNA was extracted from the sections using Allprep DNA/RNA FFPE Kit (Qiagen) or GenElute FFPE RNA/DNA Purification Plus Kit (Sigma-Aldrich) according to the manufacturer’s protocol. RNA-seq of the acquired samples was performed by the Center for Translational Genomics in Lund. The sample preparation was done according to the TruSeq Stranded mRNA Sample Preparation Guide (Part #15031047 Rev. E), and sequencing was performed using the NovaSeq 6000 System (Illumina, Inc.) following the NovaSeq 6000 Sequencing System Guide (Document #1000000019358 v11). RNA-seq data were successfully obtained from all patients except two due to low RNA yield.

Bulk RNA-seq samples of lung cancer brain metastases from the 25 patients including data for 60,662 transcripts was imported into R Studio using the DESeq2 package (27) and converted to an ExpressionSet object. In the first deconvolution, comprising of the four patients from the GeoMx study, we used the reference datasets published by Kim and colleagues (17) and Gonzalez and colleagues (24) as before. In the second deconvolution, we used our dataset GeoMx segments as the reference. For this deconvolution, the I ROIs in the GeoMx dataset were grouped by their signature (I-ECD8^+^T/Pc/Mc, I-ECD8^+^T/Pc/P, I-Fb/Mc/P, I-FoB/ECD8^+^T/NK, and I-Mc), whereas the TI ROIs were grouped according to which I ROI they bordered (TI-ECD8^+^T/Pc/Mc, TI-ECD8^+^T/Pc/P, TI-Fb/Mc/P, and TI-FoB/ECD8^+^T/NK). Segments labeled as “TI-Unknown” or “TC” were also included. Two TI ROIs which bordered both I-Fb/Mc/P and I-ECD8^+^T/Pc/Mc were omitted, bringing the number of reference segments down to 94.

For both deconvolutions, the “SCDC_prop” function from the SCDC package was used to deconvolute the bulk RNA samples using either the Kim and colleagues, the Gonzalez and colleagues, or the GeoMx dataset as a reference (28). In cases in which a patient had more than one sample, the sample with the lowest ID was kept.

As before, the “survdiff” function from the R package “survival” was used to calculate differences in survival based on the GeoMx deconvolution. Patients with a measured CD45^+^ infiltration lower than 3.5% were omitted from the cohort. The patients were then stratified into two groups by the median TI-Fb/Mc/P abundance (33.5%). After calculation of the median, patients with an unknown time to death were omitted from the survival analysis.

### Multiplex IHC

We used the Solid Tumor Immunology 6-Plex Panel (OP7TL4001KT, Akoya Biosciences) for multiplex IHC (mIHC). This is a preoptimized kit that provides paired antibodies, their staining order, suggested concentration, and the relative Opal fluorophore pairing for the detection. Additional information on the antibodies used in the panels, such as concentrations and article numbers, can be found in Supplementary Table S3.

Tissue slides were baked at 60°C overnight. The tissue was rehydrated with xylene followed by an ethanol gradient. The tissue was fixed with 10% neutral buffered formalin for 20 minutes at room temperature and washed. Next, the slides were exposed to cycles of incubations with different primary antibodies. Before applying each antibody, the slides were rinsed with an AR6 or AR9 buffer solution depending on the epitope being marked, and antigen retrieval was done using a pressure cooker (Retriever 2100, Aptum Biologics Ltd.) before every staining. This also served to quench endogenous peroxidases and remove antibodies from earlier staining. The slides were rinsed in Tris-buffered saline with 0.1% Tween (TBST) and incubated with blocking solution for 10 minutes at room temperature. Next, the slides were incubated in a humidified chamber with the primary antibody for 30 minutes, horseradish peroxidase–conjugated secondary antibodies (RRID: AB_2890927) for 10 minutes, and the Opal fluorophore for 10 or 30 minutes, depending on the primary antibody. The slides were washed with TBST three times for 2 minutes each between incubations. After the cycle had been repeated for each antibody, the slides were pressure-cooked in AR6 solution, rinsed in distilled water and TBST, and incubated with 4′,6-diamidino-2-phenylindole solution at room temperature for 5 minutes. After a final wash in TBST and distilled water, the slides were mounted.

The stained tissue and the spectral library were scanned using the PhenoImager HT device (Akoya Biosciences, Inc.). Tissue stamps corresponding to the TMA cores were selected in PhenoChart (RRID: SCR_019156), followed by unmixing and spatial analysis in InForm (also from Akoya Biosciences, RRID: SCR_019155). Sample tissue for lung cancer brain metastases was used to prepare a spectral library for unmixing the signals in the scanned images of the mIHC staining.

### mIHC data analysis

The scanned image files were processed in the InForm 2.6 software in which they were subject to fluorophore unmixing, tissue segmentation, cell segmentation, and cell phenotyping. Areas of the core with folded or damaged tissue were ignored in these steps. The software lets the user label tumor and stroma regions and the various cell phenotypes in the images. The software then uses machine learning to create an algorithm that can be applied across the cores. Pan-CK signal was used to train the tissue segmentation algorithm, whereas cell segmentation was performed using Pan-CK and 4′,6-diamidino-2-phenylindole signals. Phenotypes were assigned in InForm using the intensity of the fluorescent Opal fluorophores. The data exported from InForm were analyzed in R Studio using the packages phenoptrReports and SPIAT (29, 30). For each patient, only cell populations with a stroma prevalence of at least 0.50% in at least one of the slides were included when reporting percentages. Cells with unconventional phenotypes (such as CD20^+^CD68^+^ cells) or positive for more than two antibodies were not reported in the results but were considered when calculating percentages of the total cell count (Supplementary Table S4).

The cells in neighborhood (CIN) metric was calculated using the “average_percentage_of_cells_within_radius” function from the SPIAT R package, whereas the normalized mixing score (NMS) was calculated using the “mixing_score_summary.” If a cell type was missing from a core, then the CIN and NMS values for this cell type could not be calculated for that core. Thus, when calculating means for each patient, only cores that included this cell type were included. We used a search radius of 30 μm for both metrics.

To calculate the distances from immune cells to their nearest MTC, we used the “calculate_minimum_distances_between_celltypes” function in SPIAT. Areas of the core with separation artefacts and folded or damaged tissue were ignored from the analysis.

Counts within radius and nearest neighbor distances were calculated using the “nearest_neighbor_summary” and “count_within_summary” functions from the phenoptrReports R package.

### Survival analysis

RNA-seq data were downloaded from a study which had collected 55 RNA samples from 48 patients with brain metastasis (of which 28 samples were from patients with lung cancer) and measured expression levels for 730 immune-related and 40 additional housekeeping genes (31). One sample was removed as the overall survival (OS) time was missing, and three further samples were removed as they were the second sample from the same patient, leaving 24 patients with one sample each. The median age of the patients was 59, with a range spanning 44 to 79 years of age (interquartile range: 50–70 years old). As before, the SCDC package was used to deconvolute these samples using our GeoMx dataset as a reference, using only the 485 genes which were present in both datasets for the computation. Differences in survival were calculated using the R package “survival.”

RNA-Seq V2 RSEM gene expression data and clinical data from 517 samples of primary lung cancer in the Firehouse Legacy cohort was downloaded from The Cancer Genome Atlas (TCGA) through the cBioPortal for Cancer Genomics (32–34). Nine samples were removed as the OS time was missing, and two further samples were removed as they were the second sample from the same patient, leaving 506 patients with one sample each. The median age of the patients was 66, similar to our cohort, with a range of 38 to 88 years of age (IQR: 59–73 years old). The most common cancer stage in the cohort was stage I (*n* = 271), followed by stage II (*n* = 120), stage III (*n* = 81), and stage IV (*n* = 26). Of the total patients, 53.6% were female. The deconvolution was performed using SCDC using all genes as reference, and differences in survival were modeled using “survival.”

### Statistics

For the GeoMx analysis, log_2_FCs and two-sided *P* values for the DE analysis between ROIs were calculated using the “mixedModelDE” function from the GeomxTools package (RRID: SCR_023424; ref. 14), specifying the type of region as a fixed effect and the patient ID as a random effect. The logarithm in base 2 of the Q3-normalized counts were used an input data for these analyses (see “Spatial transcriptomics data analysis”for detailed information on how the normalization was carried out). The FDRs (“*q*-values”) were calculated using the qvalue package in R (RRID: SCR_001073; ref. 35), which fixes a rejection region, infers the number of true null hypotheses, and finally attempts to estimate the type I error rate (36). If multiple immune signatures or multiple patients were being compared at the same time, the *q*-values were calculated across all comparisons. Genes with a two-tailed *P* value less than 0.05 and a *q-*value less than 0.10 were considered significant. The log_2_FC cutoff was set to 0.75 for the I-associated TI ROI DE analysis and 0.50 for the per-patient TC–TI analysis. The bioinformatic analyses were performed blinded to the patient characteristics.

When running the aforementioned GSEA tests in clusterProfiler on differentially expressed genes between signatures of I ROIs, or on differentially expressed genes between TC and TI ROIs, a two-tailed *P* value cutoff of 0.05 as well as a FDR cutoff of 0.10 was for discovered ontologies. Other values were kept to their defaults.

Owing to the low number of tissue samples used in the mIHC analysis, no inferential statistics were performed. Metrics from the same sample (such as the nearest neighbor distance) were reported as the median, whereas aggregated metrics for multiple samples of the same patient were reported as the mean of the samples.

### Data availability

Research data supporting this publication will be made available on the European Genome–Phenome Archive. All raw datasets are available from the corresponding author upon request in compliance with Swedish legislation.

The code used to analyze the data in this study is also available from the corresponding author upon request.

## Results

### Immune infiltration in brain metastasis is associated with worse survival

**Figure 1:**
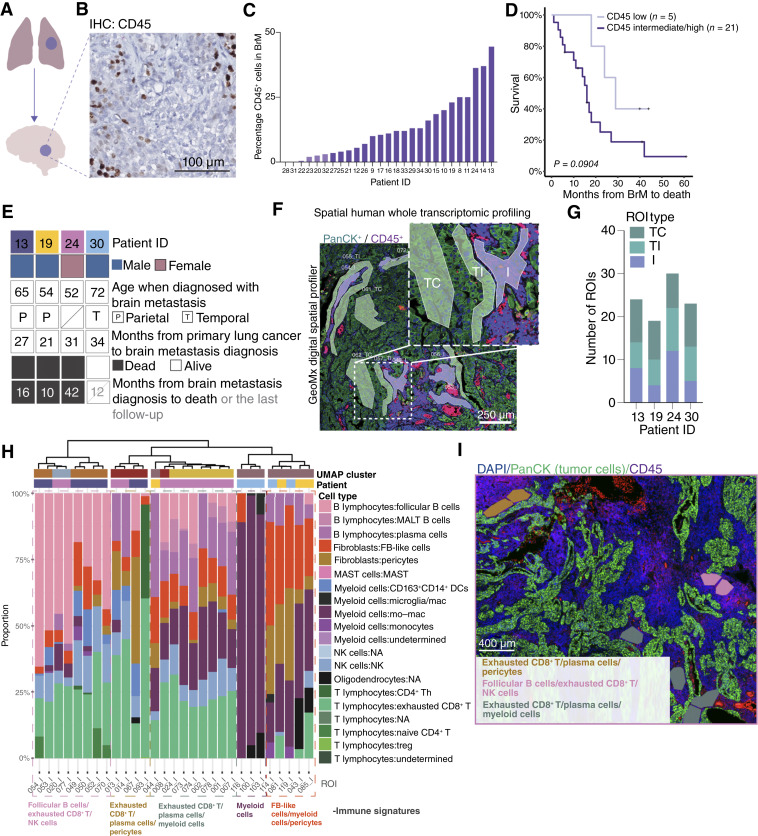
Immune landscape in lung cancer brain metastases. **A,** Schematic illustration of the sampling. **B,** IHC staining of lung cancer brain metastasis with anti-CD45 antibody. **C,** Distribution of percentage of CD45^+^ cells for the lung cancer brain metastasis samples. **D,** Kaplan–Meier curve of OS from the moment of brain metastasis diagnosis for the lung cancer brain metastasis cohort, split into two groups by the 25th percentile of CD45^+^ infiltration. *P* value calculated according to the log-rank test. **E,** Patient characteristics for the four lung cancer patients with brain metastases. **F,** Micrograph sample from the NanoString GeoMx Digital Spatial Profiler depicting ROIs marked for mRNA collection. **G,** Number of ROIs per ROI type and patient in the GeoMx dataset after quality control. **H,** Estimated cell proportions in the I ROIs according to the spatial deconvolution result. The I ROIs have been clustered into five immune signatures. **I,** Location of patient 24’s I ROIs on their source microscope slide. BrM, brain metastasis.

Immune cells constitute a significant barrier to metastatic outgrowth (37). However, they can also be influenced by secreted cytokines from tumor cells to acquire protumoral and prometastatic functions (38, 39). To determine the prognostic role of the immune landscape in lung cancer brain metastases, we first stained a lung cancer brain metastasis TMA with the pan-leukocyte marker CD45 (Fig. 1A–C). The histologic and molecular characteristics of the patients’ primary tumors and brain metastases, as well as details on pre– and post–brain metastasis treatment, can be viewed in Supplementary Table S1. Interestingly, patients with intermediate or high infiltration of immune cells (CD45^+^ cells) had worse survival after brain metastasis (Fig. 1D; Supplementary Fig. S1A), contradicting the literature detailing the positive prognostic role of CD3^+^, CD8^+^, and CD45RO^+^ lymphocytes in lung, breast, melanoma, and renal cell brain metastases, among others (40, 41).

### Spatial WTA reveals recurring intratumoral immune signatures in lung cancer brain metastases

The unexpected effect of immune cell infiltration in lung cancer brain metastases warranted further investigation into the immune landscape. Although previous studies have generated a comprehensive overview of immune cell populations in human brain metastases (12, 13, 17, 24, 42), they have rarely provided information on the spatial organization of different cells and their prognostic impact. In this study, we aimed at investigating spatial cellular networks in more detail.

To this end, we performed spatial WTA profiling with an extensive multiregional sampling of brain metastases from four patients with lung cancer (Fig. 1E). Two of the patients (#19 and #24) had previously been treated with cisplatin and the *Vinca* alkaloid vinorelbine; the remainder had only been treated with surgical removal of the primary tumor with or without radiation therapy (Supplementary Table S1). In total, we annotated 121 ROIs over the four samples, including regions with immune cell infiltrates (I), MTCs at the TI interface, and MTCs in the TC (Fig. 1F). This focused approach allowed us to prioritize the generation of highly detailed representations of the tissue architecture for each sample. After quality control and data preprocessing, each brain metastasis sample had between 19 and 30 ROIs (Fig. 1G). An unsupervised UMAP plot over the retained ROIs showed that the tumor ROIs clustered within each patient, whereas their immune ROIs clustered together (Supplementary Fig. S2A). The notable exception was one I ROI from patient 30 (“103_I”), which clustered together with the tumor ROIs from that patient. A closer examination of the slide scan revealed pan-cytokeratin signal in the ROI (Supplementary Fig. S2B), suggesting that this ROI may have included MTCs, and by extension, tumor-expressed genes.

To annotate the cell types present in the immune infiltrates, we performed spatial deconvolution on the 29 I ROIs (15). The analysis estimated the relative cell abundances in each I ROI based on a reference dataset, omitting MTCs. We used two reference datasets consisting of scRNA-seq data from human lung cancer brain metastases (17, 24). Both revealed five groups of immune infiltrate signatures (Fig. 1H; Supplementary Figs. S2 and S3). Two of the five immune signatures were prominently associated with one patient. However, 80% (four of five) of the immune signatures were observed in more than one patient (Fig. 1H). A female patient, patient 24, had the most heterogeneous immune landscape, albeit in spatially segregated compartments (Fig. 1I).

The deconvolution using a reference dataset by Kim and colleagues (17) showed a first group of three I ROIs almost entirely composed of myeloid cells (Mc, Fig. 1H). Similarly, the deconvolution using the reference dataset by Gonzalez and colleagues (24) revealed a group of ROIs dominated by APOE^+^ metastasis-associated macrophages (Supplementary Fig. S3B). Using the Kim and colleagues dataset, a second group of I ROIs was predicted to have fibroblast-like cells, myeloid cells, and pericytes (Fb/Mc/P). The third group defined by this dataset contained a large abundance of follicular B cells, exhausted CD8^+^ T cells, and NK cells (FoB/ECD8^+^T/NK). The fourth and fifth groups were dominated by exhausted CD8^+^ T cell and plasma cell signatures: of these, the former (ECD8^+^T/Pc/P) had a stronger pericyte signature, whereas the latter had a stronger myeloid signature (ECD8^+^T/Pc/Mc; Fig. 1H). The latter was also the most common immune signature, with 9 of the 29 I ROIs being assigned to it, and was most prevalent in patient 24 in whom it accounted for 8 of the 12 I ROIs. Six of eight I ROIs from patient 13 displayed the FoB/ECD8^+^T/NK and ECD8^+^T/P/Pc signatures. Three of four I ROIs from patient 19 featured an Fb/Mc/P signature, whereas patient 30 had three I ROIs with a Mc signature and two with an Fb/Mc/P signature.

To validate the findings from SpatialDecon, we performed RNA deconvolution on the bulk RNA-seq data from the patients in our lung cancer brain metastasis cohort using both the Kim and colleagues and Gonzalez and colleagues datasets as reference. Unfortunately, the bulk RNA deconvolution using the Kim and colleagues reference was dominated by oligodendrocyte fractions and failed to recapitulate immune cell populations such as T and B cells, perhaps owing to their paucity compared with brain and cancer cells (Supplementary Fig. S4A). Similarly, the deconvolution using the Gonzalez and colleagues reference was dominated by astrocyte and endothelial cell signatures (Supplementary Fig. S4B). This illustrates the limitation of bulk RNA-seq, hindering precise delineation of rare cell populations.

Collectively, our well-annotated and extensive multiregional sampling of lung cancer brain metastases revealed heterogeneous immune signatures within most patients which, however, recurred across different patients.

### mIHC highlights lymphoid and myeloid segregation

**Figure 2:**
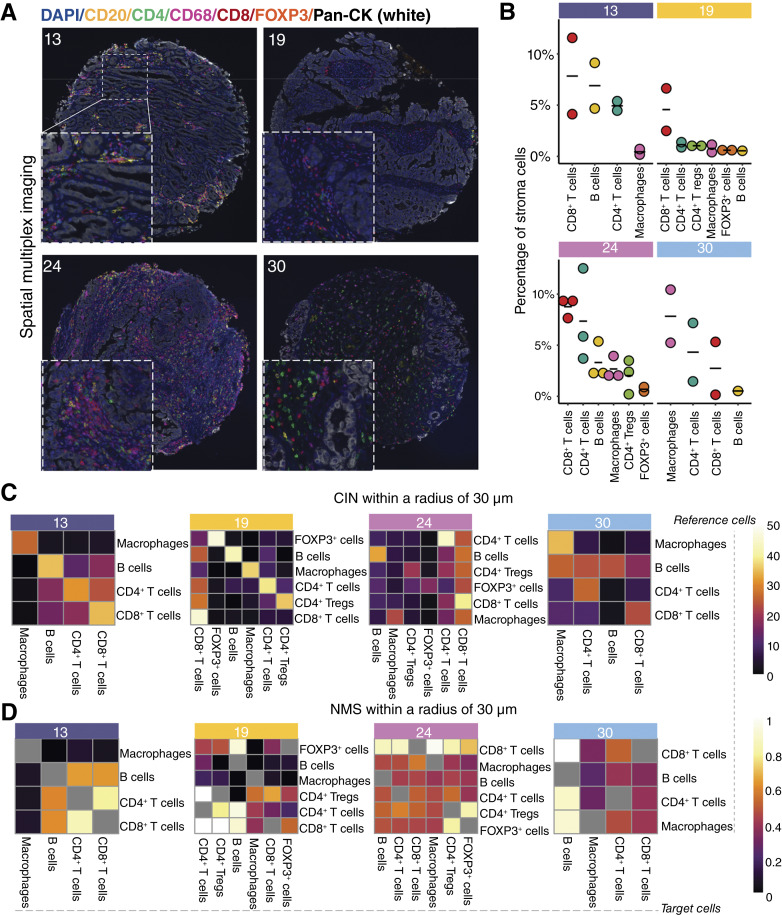
Lymphoid and myeloid segregation. **A,** Representative images depicting one TC for each of the four patients. The cores have been stained for CD20 (B cells, in yellow), CD4 (CD4^+^ T cells, in green), CD8 (CD8^+^ T cells, in red), CD68 (macrophages, in magenta), FOXP3 (regulatory T cells, in orange), Pan-CK (MTCs, in white), and 4′,6-diamidino-2-phenylindole (nuclei, in blue). **B,** Abundances of immune cell populations in stroma regions are shown as a percentage of all stroma cells. Each dot corresponds to one TC (*n* = 9). **C,** Mean percentage of CIN between reference (*y*-axis) and target (*x*-axis) populations across the whole core (tumor and stroma) for each patient, using a search radius of 30 μm. *n* = 2 applies to all observations except for patient 24, in which case *n* = 3. **D,** NMSs for reference (*y*-axis) and target (*x*-axis) populations for the whole core. The values are displayed as a mean of the different TCs and a search radius of 30 μm. Gray squares indicate that the NMS was unable to be calculated. DAPI, diamidino-2-phenylindole.

To extend our spatial analyses of immune infiltrates in lung cancer brain metastases with an orthogonal approach, we performed mIHC staining on TCs from the same patients, on which we stained with five immune cell markers: CD4 (T-helper cells), CD8 (cytotoxic T cells), CD20 (B cells), FOXP3 (regulatory T cells, T_regs_) and CD68 (macrophages; Fig. 2A). These microscope slides were then scanned using a fluorescence microscope.

We used InForm, an image analysis software developed by Akoya Biosciences, to identify 56,011 single cells in the images, of which 29,500 were in stroma and 26,501 were in tumor regions. The counts of all assigned phenotype combinations are listed in Supplementary Table S4. For downstream analyses, we chose to focus on B cells, CD4^+^ T cells, CD8^+^ T cells, FOXP3^+^CD4^+^ regulatory T cells, single-positive FOXP3^+^ cells, macrophages, and MTCs (PanCK^+^). These established cell types had a prevalence of at least 0.50% in stroma areas in at least one TC. Three other cell types passed this threshold but were excluded from further analyses as they featured unconventional phenotypes (CD4^+^CD68^+^ cells, CD8^+^CD20^+^ cells, and CD4^+^PanCK^+^ cells), likely a consequence of tissue artefact in the analytical framework. In total, 31,667 of the identified cells were included in downstream analyses, 23,715 were excluded as they were negative for all markers, and 629 were excluded due to insufficient prevalence or unconventional phenotype. We aggregated the number of included cell types per sample (Supplementary Fig. S5).

Then, we calculated the percentage of each cell type population within the stroma (Fig. 2B). CD8^+^ T cells were the most prevalent immune cell type for all patients except for patient 30. The analysis confirmed the higher prevalence of CD8^+^ T cells and B cells in the stroma of patients 13 and 24 compared with patients 19 and 30. Macrophages were the most frequent immune cell type in patient 30’s stroma regions, further corroborating the spatial deconvolution results. Surprisingly, patient 19 had a large CD8^+^ T cell population when the spatial deconvolution had only inferred strong stromal and myeloid cell populations. T_regs_ only surpassed 0.50% of stroma cells in patients 19 and 24 (reaching 1.18% in patient 19 and 3.49% in patient 24) but accounted for at most 0.48% of the stroma for patients 13 and 30.

We next analyzed spatial relationships between immune cell populations to understand their organization within brain metastases (Fig. 2C and D; Supplementary Fig. S6). Using SPIAT (30), we performed a CIN analysis. CIN is the number of target cells as a percentage of all cells around a given radius of a reference cell type averaged for all reference cells. We used a search radius of 30 μm, equal to the diameter of four lymphocytes (43), to calculate the CIN values. The analysis revealed that macrophages had low percentages of other cell types in their neighborhoods, indicating spatial exclusion. B cells often had high percentages of CD4^+^ and CD8^+^ T cells in their neighborhoods, indicating colocalization. CD4^+^ T cells had very few macrophages in their neighborhoods but modest amounts of CD8^+^ T cells. T_regs_ tended to have modest percentages of CD4^+^ and CD8^+^ T cells in their neighborhood. Overall, CD8^+^ T cells were ubiquitous in the neighborhoods of other cells except macrophages (Fig. 2C).

Local and global variations in cell density can undoubtedly affect counts of cell distribution. Thus, we used SPIAT to calculate the NMS, a colocalization metric that quantifies the number of interactions between two cell types and adjusts for the number of reference cells. The NMS analysis found that CD8^+^ T cells frequently infiltrate the neighborhoods of B cells, CD4^+^ T cells, and CD4^+^ T_regs_ (Fig. 2D). CD4^+^ T cells infiltrated neighborhoods belonging to B cells and CD4^+^ T_regs_. CD4^+^ T_regs_ infiltrated neighborhoods around CD4^+^ T cells. B cells infiltrated neighborhoods around CD4^+^ T cells and CD4^+^ T_regs_. Similar to the results from the CIN calculation, macrophages trended toward a low infiltration of other immune neighborhoods. Thus, the normalized mixing score reinforced the notion that macrophages occupy different compartments from lymphoid cells in lung cancer brain metastases.

In conclusion, the spatial single-cell analysis of immune cell populations reflected the proportions from the WTA analysis and revealed segregation between lymphoid cells and macrophages, implying distinct immune–MTC networks.

### Ligand–target analysis predicts distinct cytokine networks in lymphoid and myeloid infiltrates

**Figure 3:**
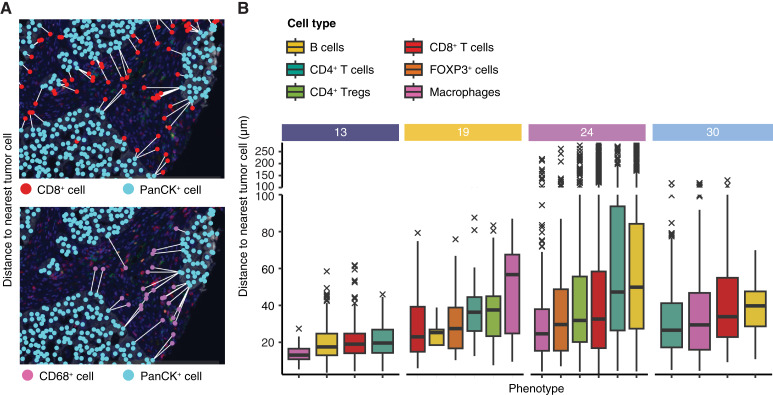
Immune and metastatic tumor cell interactions. **A,** Representative images showing the nearest MTC to each CD8^+^ and CD68^+^ cell visualized using PhenoptrReports. **B,** Boxplot of distance to nearest MTC for each immune cell calculated using SPIAT, grouped by immune cell type and patient. *n* = 2 applies to all patients except for patient 24, in which case *n* = 3. The central line of the boxplot includes the median; the boundaries of the box correspond to the first and third quartiles (25th and 75th percentiles). The length of the whisker corresponds to at most 1.5 multiplied by the IQR, measured from the edge of the hinge. Outliers are shown as crosses.

To see if MTCs contribute to intratumor immune heterogeneity and to resolve immune–MTC communications, we used SPIAT to calculate the minimum distance between each immune cell type and the nearest MTC (Fig. 3) and found numerous interpatient differences. For example, macrophages were the closest immune cell population to MTCs in patients 13 and 24 but the furthest in patient 19. Although patients 19 and 30 featured the same immune infiltrate signatures, the two closest immune cell populations to their MTCs in patient 19 (i.e., CD8^+^ T cells and B cells) were the furthest away from MTCs in patient 30. Hence, there was no consistency in the nearest MTC distances, leading us to hypothesize that the evident interpatient MTC heterogeneity (Supplementary Fig. S2A) might drive the variation.

**Figure 4:**
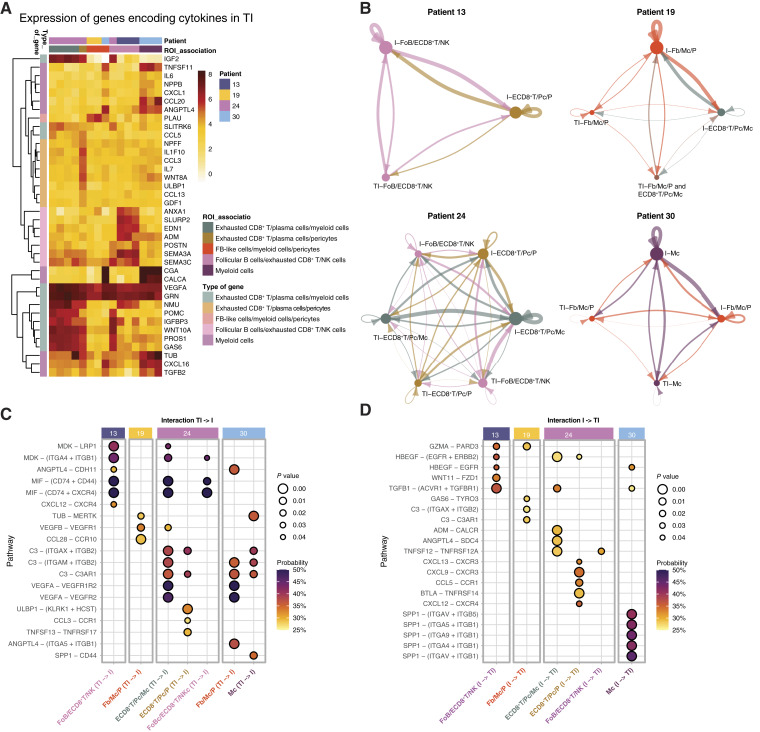
Immune and metastatic tumor cell communication. **A,** Normalized gene expression for differentially expressed cytokines for each group of I-associated TI ROIs. Significant upregulation was defined as a log_2_FC of at least +0.75 and a *q*-value of less than 0.10. **B,** Plot of significant connections of inferred secreted signaling pathways between I and TI ROIs, weighted by communication probability. TI ROIs without an associated I ROI were excluded from the plot. **C,** Dot plot of the five TI to I interactions with the highest inferred probability per patient. **D,** Dot plot of the five I to TI interactions with the highest inferred probability per patient.

To find an explanation for the lymphoid and myeloid segregation, we analyzed our annotated spatial whole transcriptomic dataset (Fig. 4A). Because the location and cell programs of the immune infiltrates could be influenced by secreted cytokines, we performed DE analysis on cytokines using the list of cytokines retrieved from the CellChat R package (22, 23). The TI ROIs were annotated with the identity of their nearest I ROI, revealing differences in gene expression between the five TI groups (Fig. 4A; Supplementary Fig. S7; Supplementary Table S5). ECD8^+^T/Pc/Mc-associated TI ROIs expressed cytokine genes such as *IGF2* and *POMC*, whereas ECD8^+^T/Pc/P-associated TI ROIs expressed *IL1F10*, *CCL3*, and *IL7*. FoB/ECD8^+^T/NK-associated TI ROIs expressed cytokine genes including *ANXA1*, *SLURP2*, and *EDN1*. Fb/Mc/P-associated TI ROIs had a high expression on *PLAU*, whereas the Mc-associated TI ROIs had high expression of, *CGA*, *CALCA*, and *CXCL16*.

We also inferred communication pathways between the TI ROIs and all I ROIs and *vice versa* using CellChat analyses (Fig. 4B; Supplementary Table S6). In line with our previous observations, we detected strong signaling between ECD8^+^T/Pc/Mc and corresponding TI ROIs for patient 24. As the coordinates of the ROIs were not considered in the analysis, unexpected interactions between I ROIs marked with one signature and distant TI ROIs annotated to a different signature were also theorized.

We next narrowed our analysis to focus only on interactions between I and TI ROIs annotated to that immune signature. Among the top interactions, we identified that lymphoid I ROIs were predominantly influenced by MTC-derived midkine and macrophage migration inhibitory factor signaling via predicted binding to LRP1/ITGA4 + ITGB1 and CD74 + CD44/CD74 + CXCR4, respectively, whereas the fibroblast/myeloid/pericyte infiltrates were influenced by ANGPTL4, SPP1, and C3-mediated complement signaling via predicted binding to ITGA5 + ITGB1, CD44, and C3AR1/ITGAM + ITGB2, respectively (Fig. 4C).

Concerning the impact of immune infiltrates on MTCs, the analysis revealed that four of the five immune groups signaled to the EGFR receptor, mostly through HBEGF. TGFB1 signaling to ACVR1 + TGFBR1 receptors was found in three groups. However, immune signature–specific signaling was also found, with ligands and ligand–receptor pairs similar to that of tumor-to-immune interactions. Fb/Mc/P in patient 19 signaled with C3–C3AR1, and the Mc infiltrates in patient 30 signaled through SPP1 (Fig. 4D).

Taken together, the lymphoid and fibroblast/myeloid/pericyte-dominated infiltrates had distinct cell–cell communications, but they also shared cytokine networks. Moreover, adjacent MTCs and immune cells harbored similar cytokine profiles, indicating that they both fostered that niche.

### MTC gene expression is influenced by proximity to immune infiltrates

**Figure 5:**
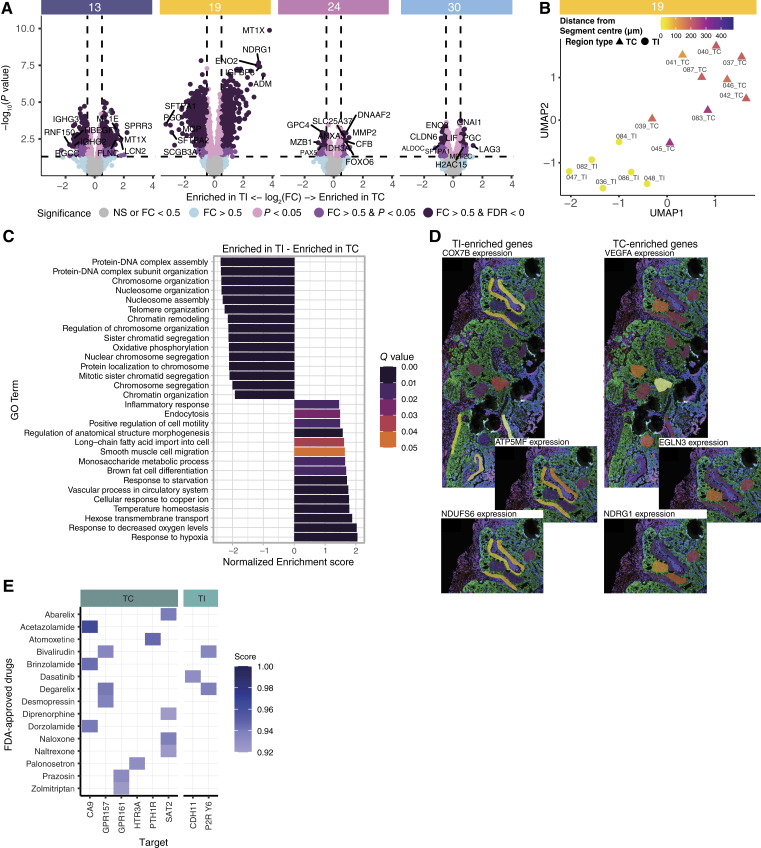
Metastatic tumor cell heterogeneity influenced by immune infiltrates. **A,** Volcano plot of differentially expressed genes between TC and TI ROIs per patient. A mean of 9.3 TC ROIs and 7.5 TI ROIs were used for each patient’s comparison. **B,** UMAP plot of the TC and TI ROIs for patient 19, with points colored by the distance between the ROI centroid center and the nearest I. **C,** Top 15 TI and top 15 TC ontologies based on a GSEA result of the differential expression result for patient 19, sorted by the normalized enrichment score. **D,** Gene expression of TI-upregulated genes and TC-upregulated genes plotted directly on the slide micrograph for patient 19. **E,** Predicted ConPLex interaction scores between FDA-approved drugs and genes from the TC/TI differential gene expression analysis. Only interactions with a score greater than 0.92 have been shown.

To see how cytokine networks may potentially influence gene expression in MTCs, we next investigated how MTCs at the immune interface (i.e., TI ROIs) differed from MTCs in the core of the metastatic lesion (i.e., TC ROIs; Supplementary Fig. S8, Supplementary Table S7). All four brain metastases presented differentially expressed genes in TI ROIs compared with TC ROIs (Fig. 5A; Supplementary Table S7). Patient 19 was the patient with the most differentially expressed genes; in fact, the TI and TC ROIs clustered to different locations on a UMAP plot based on their distance between the nearest immune infiltrate and the ROI center (Fig. 5B). Next, we conducted a GSEA on the differentially expressed genes from this patient, showing that TI ROIs upregulated genes related to oxidative phosphorylation whereas TC ROIs upregulated genes related to response to hypoxia (Fig. 5C).

To further grasp the spatial distribution of the gene expression, we plotted patient 19’s differentially expressed genes onto the scanned digital image of the WTA slide (Fig. 5D). To represent the oxidative phosphorylation genes, we chose cytochrome C oxidase subunit 7B (*COX7B*, log_2_FC = −0.59; *q* < 0.009), ATP synthase membrane subunit F (*ATP5MF*, log_2_FC = −0.77; *q* < 0.002), and NADH: ubiquinone oxidoreductase core subunit S6 (*NDUFS6*, log_2_FC = −1.05; *q* < 0.002). From the genes related to hypoxia in the DE result, we chose *VEGFA* (log_2_FC = 1.83; *q* < 0.002), Egl-9 family hypoxia-inducible factor 3 (*EGLN3*, log_2_FC = 1.82; *q* < 0.0002), and N-Myc downstream regulated 1 (*NDRG1*, log_2_FC = 3.06; *q* < 0.0002). The overlayed gene expression demonstrated the ubiquitous expression of oxidative phosphorylation genes in all TI segments and that genes related to hypoxia were instead concentrated in the core regions of the tumor.

### Computational modeling identifies candidate drugs overcoming spatial tumor heterogeneity

Building upon our spatial observations, we next leveraged this knowledge to explore potential therapeutic interventions. Considering the observed spatial tumor heterogeneity, we performed computational modeling of drug–target interactions to decipher whether any existing FDA-approved drugs could target both MTC regions (i.e., TI and TC ROIs) or whether combinational therapy should preferably be applied. We prepared a gene set consisting of 759 genes upregulated in TC ROIs in patient 19 and a TI gene list consisting of 558 genes from the same patient. We used ConPLex, a protein language model incorporating contrastive learning for computational modeling of potential drug–target interactions. This deep-learning platform can predict drug–target interactions with more than 80% accuracy (26). The ConPLex interaction score assessed the protein–molecule interactions for the TC and TI genes in patient 19 and the FDA-approved molecules. Interestingly, TC regions had more potential drug–target interactions. The analysis did, however, reveal two FDA-approved drugs that potentially target MTCs in both TI and TC regions: bivalirudin, a reversible direct thrombin inhibitor (44), and degarelix, a GnRH antagonist used in the treatment of prostate cancer (45). In our language model, they both targeted the TC-associated protein G protein–coupled receptor 157 (GPR157) and the TI-associated protein pyrimidinergic receptor P2Y6 (P2RY6; Fig. 5E).

Taken together, the comparison revealed interesting metabolic differences between the TC and TI MTCs, highlighting a hypoxic response in TC MTCs but proliferation in the presence of cellular respiration in the MTCs at the immune interface. Furthermore, some genes are implicated in protein–molecule interactions for already FDA-approved drugs and may thus represent therapeutic targets.

### Spatially identified tumor cellular network are associated with worse survival

**Figure 6:**
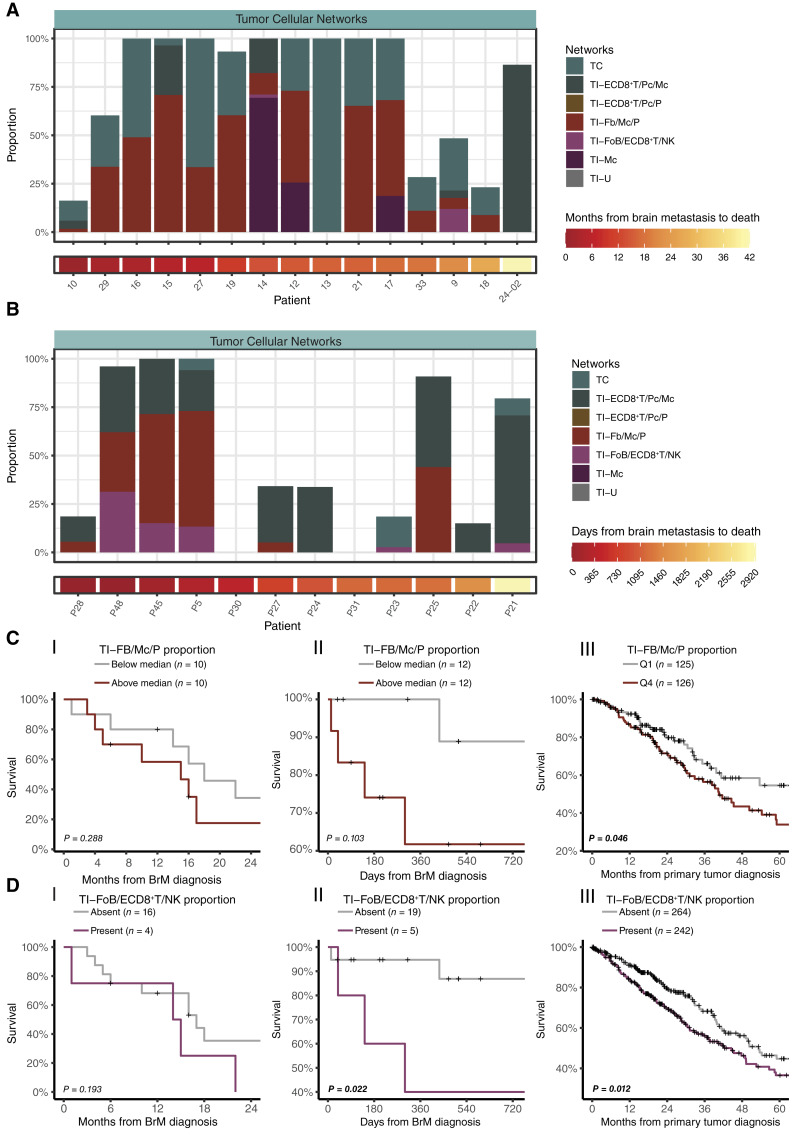
Immune–MTC networks in lung cancer brain metastases. **A,** Estimated cell proportions in the lung cancer brain metastasis dataset according to bulk RNA deconvolution with our GeoMx dataset as reference for patients with intermediate or high CD45^+^ infiltration and with known time of death. **B,** Estimated cell proportions in the Rubio-Perez dataset (31) according to bulk RNA deconvolution with our GeoMx dataset as reference. **C,** Kaplan–Meier curves illustrating survival in our lung cancer cohort (i.), the Rubio-Perez and colleagues (31) cohort (ii.), and the TCGA primary lung cancer cohort (iii.), stratified by the estimated TI-Fb/Mc/P proportion amongst the samples. *P* values were calculated according to the log-rank test. **D,** Kaplan–Meier curves illustrating survival in our lung cancer cohort (i.), the Rubio-Perez and colleagues (31) cohort (ii.), and the TCGA primary lung cancer cohort (iii.), stratified by the estimated TI-FoB/ECD8^+^T/NK proportion amongst the samples. *P* values were calculated according to the log-rank test. BrM, brain metastasis.

In an attempt to determine the clinical impact of our described immune–MTC cellular networks, we performed RNA deconvolution using our I and TI networks as reference on bulk RNA-seq data from our own lung cancer brain metastasis cohort and a cohort from a study by Rubio-Perez and colleagues (Figure 6A and B; Supplementary Figure S9; ref. 31). When viewing the estimated fractions for deceased patients with CD45^intermediate/high^ lung cancer brain metastases in our cohort as well as the deceased patients in the Rubio-Perez cohort, we noted that patients with a shorter elapsed time from brain metastasis diagnosis to death had a higher inferred fractional abundance of the TI-Fb/Mc/P tumor cellular network (Fig. 6A and B). Indeed, patients with an above-median fractional abundance of the TI-Fb/Mc/P tumor cellular network tended to succumb to their disease earlier compared with those with a lower abundance (Fig. 6C). To further validate these findings in a larger cohort, we performed bulk RNA deconvolution on a primary lung adenocarcinoma cohort (*n* = 506) obtained from TCGA. Indeed, there was a significant difference in OS between patients in the lowest and highest quartiles of the fractional abundance of the TI-Fb/Mc/P tumor signature (Fig. 6C). Of note, although the presence of the TI-FoB/ECD8^+^T/NK network had no impact on survival in our cohort, a significant association was found between the presence of this network and survival for the Rubio-Perez and TCGA cohorts (Fig. 6D).

Consequently, although it was difficult to identify immune cell networks in clinical bulk RNA data (Fig. 6A), the analysis revealed that MTCs with TI-Fb/Mc/P and TI-FoB/ECD8^+^T/NK profiles have a negative prognostic impact on survival. More importantly, these data highlight the potential of spatial technologies in identifying immune–MTC cellular networks with prognostic and therapeutic significance.

## Discussion

In this study, we used spatial whole transcriptomic profiling on extensive multiregional sampling and multiplex imaging to interrogate the immune and tumor landscapes in lung cancer brain metastases. Our analyses revealed intratumoral immune and tumor heterogeneity. We found that myeloid and lymphoid cells are influenced by distinct cytokine networks and are spatially segregated into different compartments and that MTCs in a network of fibroblast-like cells, myeloid cells, and pericytes, or a network of follicular B cells, exhausted CD8^+^ T cells, and NK cells, may have a negative prognostic effect. Despite differences between MTCs at the immune interface and in the core, we identified candidate FDA-approved drugs that potentially target both MTC entities.

Our finding that high CD45^+^ infiltration was associated with poor survival contradicts previous studies, which found no association between tumor-infiltrating lymphocytes in NSCLC brain metastases and OS (46). It also contrasts with studies that found a positive effect of tumor-infiltrating lymphocytes (40, 41). However, the intratumoral immune heterogeneity seen in our analyses corroborates previous reports claiming that multiple contrasting immune microenvironments coexist within a single tumor (47, 48). For example, spatial profiling of primary melanoma revealed the coexistence of both immunoediting and immune evasion in a single tumor (47). In primary NSCLC, the divergence of immune cell infiltration within a tumor was associated with the local mutational diversity (48). Although our study describes immune heterogeneity within each brain metastasis, we also illustrate how immune signatures recur between patients. This finding is significant as, in line with other studies (12, 17, 24, 49), it illustrates that lung cancer brain metastases may contain conserved immune signatures, or networks, which could be targets for future therapies. Interestingly, our study outlined different immune compartments with exhausted CD8^+^ T cells, which recalls a previous study showing that CD8^+^ T cells in different regions of a tumor gain different properties driven by local antigen signaling (50), once more suggesting that immune heterogeneity might be a consequence of tumor heterogeneity.

Previous studies have shown a negative correlation between T cells and tumor-associated macrophages or monocytes in human brain metastases (51). Our separation between myeloid and lymphoid infiltrates is noteworthy as it implies that immune infiltrates in brain metastases might exist on a spectrum. The observed spatial relationships might also be a consequence of T cell exclusion mediated by other cells in the tumor microenvironment (39, 52). For example, CD14^+^CD206^+^ macrophages in the brain metastasis perivascular space can potentially confine immune cells to the space around blood vessels and prevent cells such as T cells from accessing the tumor (53). The presence of fibroblast-like cells in patient 30 is noteworthy as fibrosis-related genes have been shown to be upregulated in lung cancer brain metastasis compared with control brain tissue (12). T cells and immunosuppressive bone marrow–derived macrophages preferentially localize to collagen-rich regions in brain metastases (54), whereas collagen has been shown to induce immunosuppressive macrophage polarization (55). Taken together, this suggests a potential tumor microenvironment–organizing effect of fibroblast-like cells in our sample.

The separation between myeloid and lymphoid infiltrates may also be governed by cytokines secreted from MTCs at the TI interface. For instance, we described midkine and macrophage migration inhibitory factor signaling from TI interface ROIs to their associated lymphoid immune cells, interacting with receptors such as CD74. CD74 has been associated with B-cell proliferation and survival (56) and lymph node metastasis (57). On the myeloid side, our results suggest that MTCs adjacent to myeloid (Mc) immune infiltrates may interact with myeloid cells through the SPP1–CD44 axis. *SPP1* has been correlated with poor prognosis when expressed by tumor cells (58). Likewise, SPP1 signaling to integrin receptors was also shown in the reverse direction, from myeloid cells to MTCs. *SPP1* was upregulated in a cluster of APOE^+^ metastasis macrophages in a study by Gonzalez and colleagues (24) and maintains its negative prognostic role when expressed in macrophages (59). Notably, SPP1^+^ macrophages have been shown to interact with FAP^+^ fibroblasts in colorectal cancer and can stimulate the generation of desmoplastic structures limiting T-cell infiltration (60). The observed C3-signaling and EGF receptor signaling are further pathways that may warrant additional investigation.

The highlighted cytokine cross-talk may explain the negative prognostic role of the I-Fb/Mc/P network. Vesicular pericytes and fibroblasts can together create fibrosis in brain metastases, whereas cancer-associated fibroblasts and M2-type tumor-associated macrophages are components of an immune evasive tumor brain microenvironment (12). Inflammatory cancer-associated fibroblasts have been shown to promote NSCLC brain metastasis through the MET-HGF pathway (61), and fibroblasts interact with endothelial cells to promote angiogenesis in lung adenocarcinoma brain metastases (17). Finally, fibroblasts in brain metastases have been shown to signal to myeloid cells through CD44 (42). Indeed, our CellChat analysis inferred VEGFA and VEGFB signaling from MTCs to adjacent immune cells in the I-Fb/Mc/P network. As a result, our findings emphasize the importance of fibroblast, myeloid cell, and tumor cell cross-talk in lung cancer brain metastases.

This project has investigated intratumoral immune heterogeneity and immune–MTC communications; however, it has some limitations. Although we have performed extensive multiregional sampling, the immune–MTC networks build on four patients, and it cannot be concluded that they are representative of lung cancer brain metastases. Likewise, we cannot rule out the possibility that an overlooked factor, such as the mutation profiles of the metastases, may have affected our observations. However, this study presents the most comprehensive multiregion sampling of human lung cancer brain metastases to date through its in-depth resolution of the lesions. Secondly, the deconvolution of the I ROIs was inevitably dependent on the reference dataset used: a known limitation of bulk RNA deconvolution (62). Astrocytes, the most abundant glia cell in the central nervous system (63), were absent in the Kim and colleagues dataset which were used as a reference. Conversely, although the dataset by Gonzalez and colleagues included astrocytes, it lacked oligodendrocytes. A deconvolution using a more comprehensive atlas of lung cancer brain metastases may mitigate these effects. Likewise, the IHC protocol could have explored more cell subtypes, such as fibroblasts and pericytes. Third, a limitation of this study was its consideration of spatial biology solely in two dimensions, yet bioinformatic pipelines for integrating stacks of microscope slides are on the way. Lastly, we used spatial transcriptomics on FFPE specimens, which runs the risk of being affected by the degradation of nucleic acids or artefacts in the base pair sequence (64). However, the RNA counts were obtained using probes conjugated to unique DNA-indexing oligonucleotides (digital spatial profiling barcodes) with unique molecular identifiers. This indirect approach to quantifying mRNA counts allows for greater quality control and makes the data less susceptible to formalin-induced degradation. Other spatial transcriptomics studies of lung cancer brain metastases have also used FFPE tissues (12, 13). Sudmeier and colleagues (50) performed spatial transcriptomics on brain metastases, including lung carcinoma, using fresh-frozen tissue, finding that immune cells are enriched in the peripheral region of brain metastases compared with the TC. In line with our results, the authors found lung cancer brain metastases to be well-infiltrated with CD8^+^ T cells.

In conclusion, this study underscores the segregation between myeloid and lymphoid cells in lung cancer brain metastases and their potential relation to immune exclusion. Moreover, our findings advocate for a paradigm shift from conventional therapeutic approaches focusing solely on individual genes or cell types toward targeting networks of immune and tumor cells in brain metastases. We invite future researchers to target recurring cellular networks of interacting immune and MTCs in order to generate more precise immunotherapeutic approaches.

## Supplementary Material

Supplementary Table 1 Molecular characteristics of the primary tumor and the brain metastases in our patient cohort (first sheet), as well as PDL1 expression and details of treatment for the patients in our lung cancer brain metastasis cohort (second sheet). BrM, Brain Metastasis; KRAS, Kirsten rat sarcoma virus; PDL1, Programmed death-ligand; PT, Primary Tumor; TP53, Tumor protein P53; RefSeq, NCBI Reference Sequence Database; HGCS, Guman Gene Connectome Server; HGVS, Human Genome Variation Society; c, reference sequence based on a protein coding RNA; p, reference sequence based on a protein (amino acid) sequence.

Supplementary Table 2 Descriptive statistics of the distances from each tumor core (TC) region of interest and the nearest immune infiltrate, measured using QuPath on the digital scan of the microscope slide. All distances are given in micrometers.

Supplementary Table 3 Antibody and fluorophore concentrations and incubation times for the multiplex immunohistochemistry protocol.

Supplementary Table 4 Count of identified phenotypes from the multiplex immunohistochemistry experiment, with proportions as a percentage of all identified cells.

Supplementary Table 5 Differentially expressed genes (first sheet) and cytokine genes (second sheet) from each tumor cell network with log2-fold changes, p-values, q-values calculated within each network and q-values calculated across all comparisons.

Supplementary Table 6 Predicted ligand-target interactions between immune and MTC networks across all networks (first sheet), adjacent networks (second sheet), immune networks including those not spatially adjacent (third sheet) and interactions plotted in Fig. 4c-d (fourth sheet).

Supplementary Table 7 Differentially expressed genes between all TI ROIs and TC ROIs with log2-fold change, p-value and q-value, for comparison with all patients (first sheet) as well as comparison per patient (second sheet).

Supplementary Figure S1 S1. Prognostic Effect of CD45+ Infiltration in Lung Cancer Brain Metastases.

Supplementary Figure S2 S2. The immune landscape in lung cancer brain metastases.

Supplementary Figure S3 S3. Analysis of the immune landscape in human lung cancer brain metastases using the second reference dataset.

Supplementary Figure S4 S4. Immune signatures in the lung cancer brain metastasis cohort.

Supplementary Figure S5 S5. Comprehensive cell counts from the multiplex immunohistochemistry analysis.

Supplementary Figure S6 S6. Calculations of spatial colocalization metrics using the phenoptrReports package.

Supplementary Figure S7 S7. Differentially expressed (DE) genes for the immune infiltrate-associated TI ROIs.

Supplementary Figure S8 S8. Differentially Expressed Genes between TI and TC ROIs.

Supplementary Figure S9 S9. Immune-MTC cellular Networks in Lung Cancer Brain Metastasis.
