# Supplementary material for: Spatial Multiomics Reveals Intratumoral Immune Heterogeneity with Distinct Cytokine Networks in Lung Cancer Brain Metastases
Source: Cancer Res Commun. 2024 Nov 6;4(11):2888–902. doi: 10.1158/2767-9764.CRC-24-0201 (PMC11539001; doi:10.1158/2767-9764.CRC-24-0201)
Supplement: Supplementary Figure S1 — S1. Prognostic Effect of CD45+ Infiltration in Lung Cancer Brain Metastases. [file crc-24-0201_supplementary_figure_s1_suppsf1.pdf]

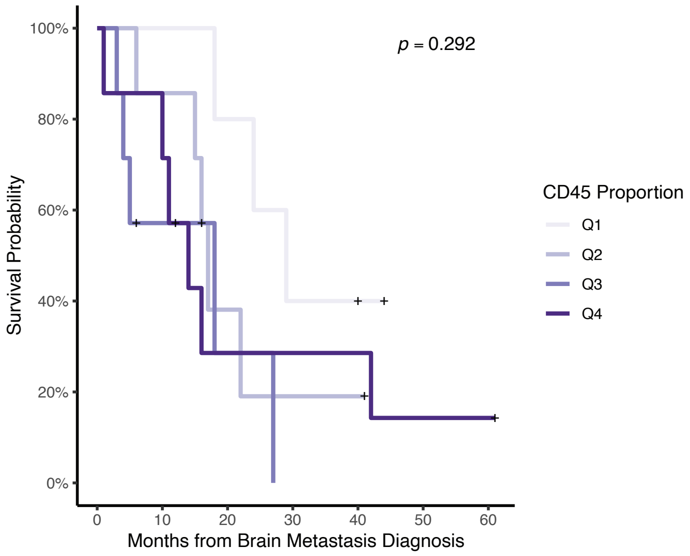

Supplementary Figure 1: Prognostic Effect of CD45+ Infiltration in Lung Cancer Brain Metastases. Kaplan-Meier curve of survival from the moment of brain metastasis diagnosis for the lung cancer brain metastasis cohort, stratified into quartiles according to the percentage of CD45+ cells in lung cancer brain metastasis.
