# Supplementary material for: Spatial Multiomics Reveals Intratumoral Immune Heterogeneity with Distinct Cytokine Networks in Lung Cancer Brain Metastases
Source: Cancer Res Commun. 2024 Nov 6;4(11):2888–902. doi: 10.1158/2767-9764.CRC-24-0201 (PMC11539001; doi:10.1158/2767-9764.CRC-24-0201)
Supplement: Supplementary Figure S2 — S2. The immune landscape in lung cancer brain metastases. [file crc-24-0201_supplementary_figure_s2_suppsf2.pdf]

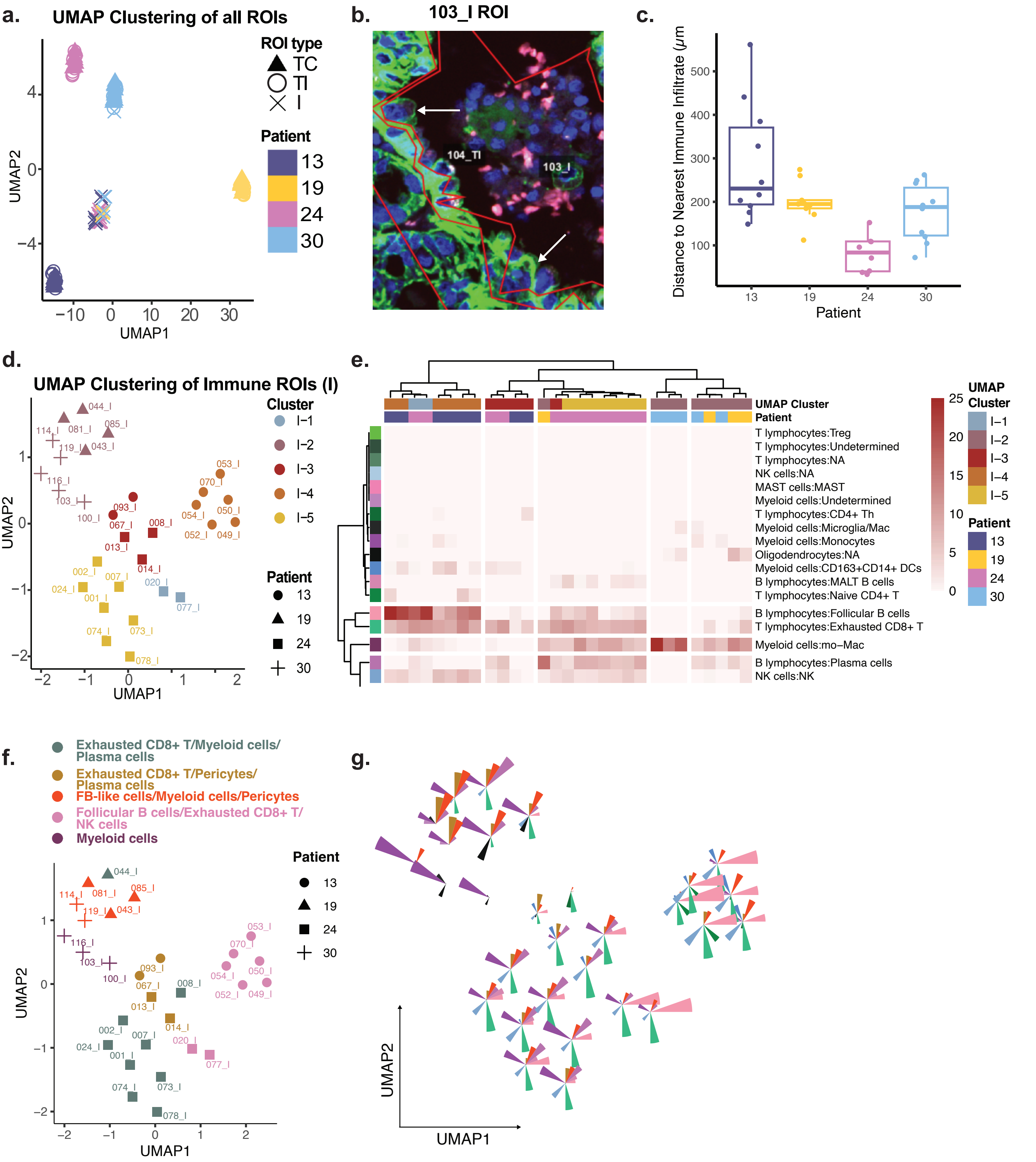

Supplementary Figure 2: The immune landscape in lung cancer brain metastases. a. UMAP plot of all regions of interest (ROIs) in the GeoMx dataset based on gene expression data. TC, tumor core ROI; TI, tumor-immune interface ROI; I, immune infiltrate ROI. **b.** Micrograph of immune-cell region of interest "103_I", indicating pan-cytokeratin signal (green) within the borders of the region of interest (red). Blue staining indicates nuclei staining with SYTO13 dye while pink indicates CD45+ signal. **c.** Boxplot of distances from each TC segment to nearest immune infiltrate as measured in QuPath, split by patient. **d**. UMAP plot of the immune infiltrate (I) ROIs from all patients, colored by cluster generated by k-means clustering. **e.** Heatmap of 'beta scores' (estimated cell abundances) of stromal cell types of the 29 immune infiltrates, calculated using the reference dataset by Kim et al[17]. **f.** Visualization of the immune ROIs, annotated according to the immune signatures, on a UMAP plot. **g.** 'Floret plot' of the I ROIs in UMAP space. The size of the 'florets' corresponds to the proportion of a particular immune cell type.
