# Supplementary material for: Spatial Multiomics Reveals Intratumoral Immune Heterogeneity with Distinct Cytokine Networks in Lung Cancer Brain Metastases
Source: Cancer Res Commun. 2024 Nov 6;4(11):2888–902. doi: 10.1158/2767-9764.CRC-24-0201 (PMC11539001; doi:10.1158/2767-9764.CRC-24-0201)
Supplement: Supplementary Figure S3 — S3. Analysis of the immune landscape in human lung cancer brain metastases using the second reference dataset. [file crc-24-0201_supplementary_figure_s3_suppsf3.pdf]

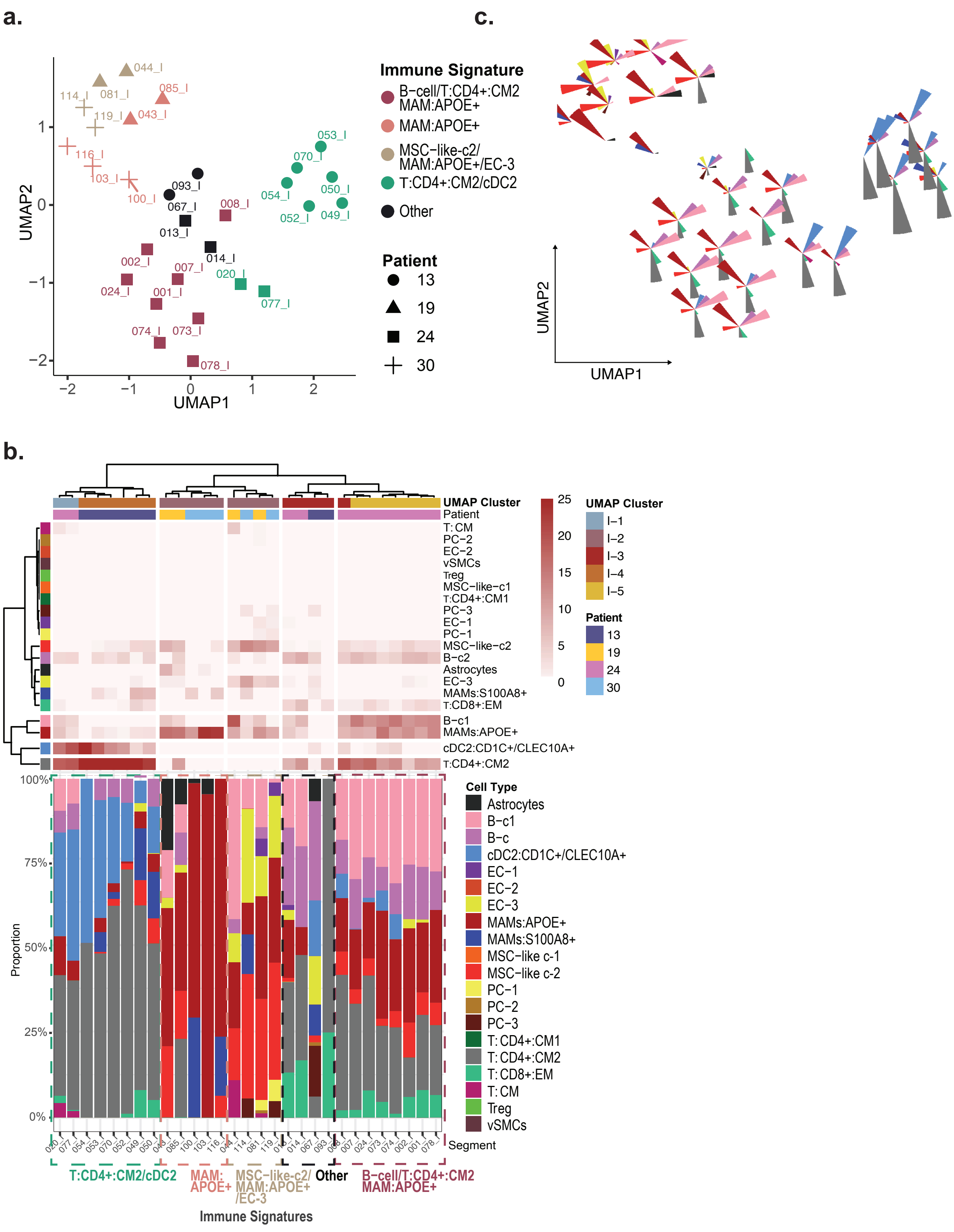


**Supplementary Figure 3: Analysis of the immune landscape in human lung cancer brain metastases using the second reference dataset[24]. a.** UMAP plot of the immune infiltrate (I) ROIs, colored by the immune signature group**. b. S**patial deconvolution results for the I ROIs. The ROIs are labeled by UMAP cluster and patient and have been split into five groups according to hierarchical clustering based on their immune signature. **c.** 'Floret plot' of the I ROIs in UMAP space. The size of the 'florets' corresponds to the proportion of a particular immune cell type.
