# Supplementary material for: Spatial Multiomics Reveals Intratumoral Immune Heterogeneity with Distinct Cytokine Networks in Lung Cancer Brain Metastases
Source: Cancer Res Commun. 2024 Nov 6;4(11):2888–902. doi: 10.1158/2767-9764.CRC-24-0201 (PMC11539001; doi:10.1158/2767-9764.CRC-24-0201)
Supplement: Supplementary Figure S4 — S4. Immune signatures in the lung cancer brain metastasis cohort. [file crc-24-0201_supplementary_figure_s4_suppsf4.pdf]

a.

b.

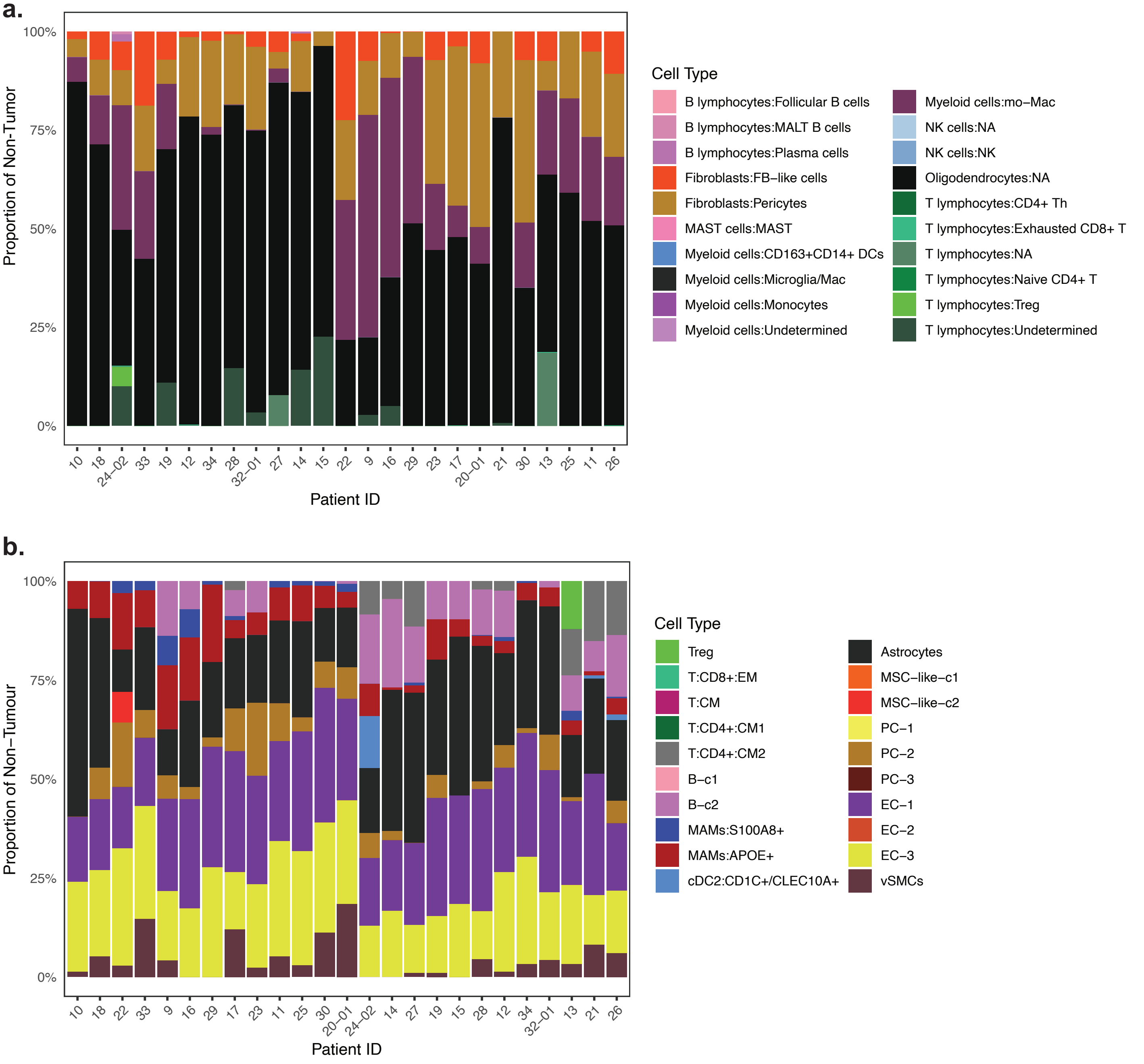

Supplementary Figure 4: Immune signatures in the lung cancer brain metastasis cohort.
a. Estimated non-tumor proportions in our lung cancer brain metastasis cohort according to the bulk RNA deconvolution using the Kim et al. cell types as reference[17]. **b.** Estimated non-tumor proportions in our lung cancer brain metastasis cohort according to the bulk RNA deconvolution using the Gonzalez et al. cell types as reference[24].
