# Supplementary material for: Spatial Multiomics Reveals Intratumoral Immune Heterogeneity with Distinct Cytokine Networks in Lung Cancer Brain Metastases
Source: Cancer Res Commun. 2024 Nov 6;4(11):2888–902. doi: 10.1158/2767-9764.CRC-24-0201 (PMC11539001; doi:10.1158/2767-9764.CRC-24-0201)
Supplement: Supplementary Figure S5 — S5. Comprehensive cell counts from the multiplex immunohistochemistry analysis. [file crc-24-0201_supplementary_figure_s5_suppsf5.pdf]

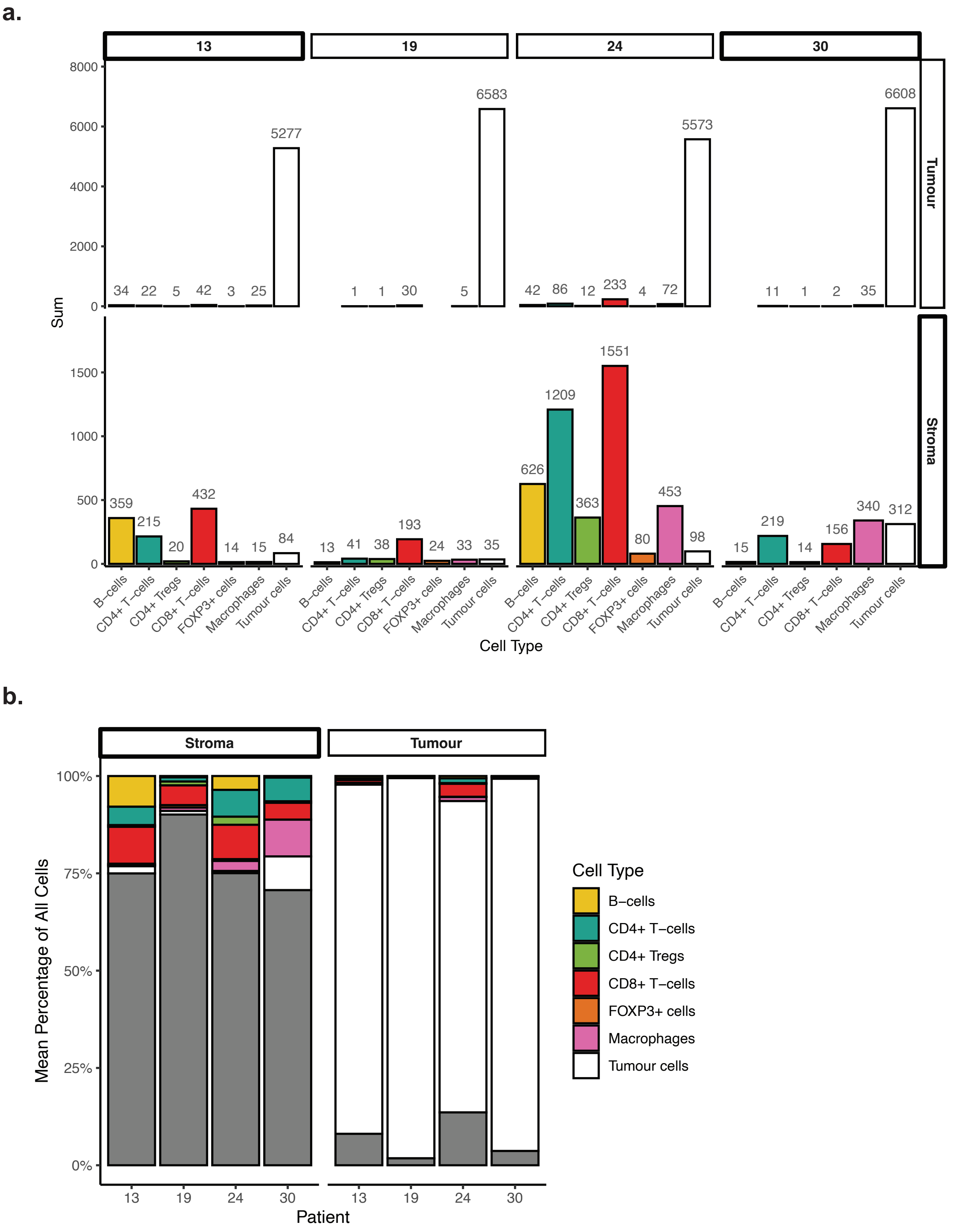

Supplementary Figure 5: Comprehensive cell counts from the multiplex immunohistochemistry analysis. **a.** Cell counts for the multiplex immunohistochemistry dataset, divided by the patient and tissue area. **b.** Number of cells as a proportion of total cells for each patient and tissue. Grey bars represent cells not included in the above phenotypes or cells that were negative for all markers.
