# Supplementary material for: Spatial Multiomics Reveals Intratumoral Immune Heterogeneity with Distinct Cytokine Networks in Lung Cancer Brain Metastases
Source: Cancer Res Commun. 2024 Nov 6;4(11):2888–902. doi: 10.1158/2767-9764.CRC-24-0201 (PMC11539001; doi:10.1158/2767-9764.CRC-24-0201)
Supplement: Supplementary Figure S6 — S6. Calculations of spatial colocalization metrics using the phenoptrReports package. [file crc-24-0201_supplementary_figure_s6_suppsf6.pdf]

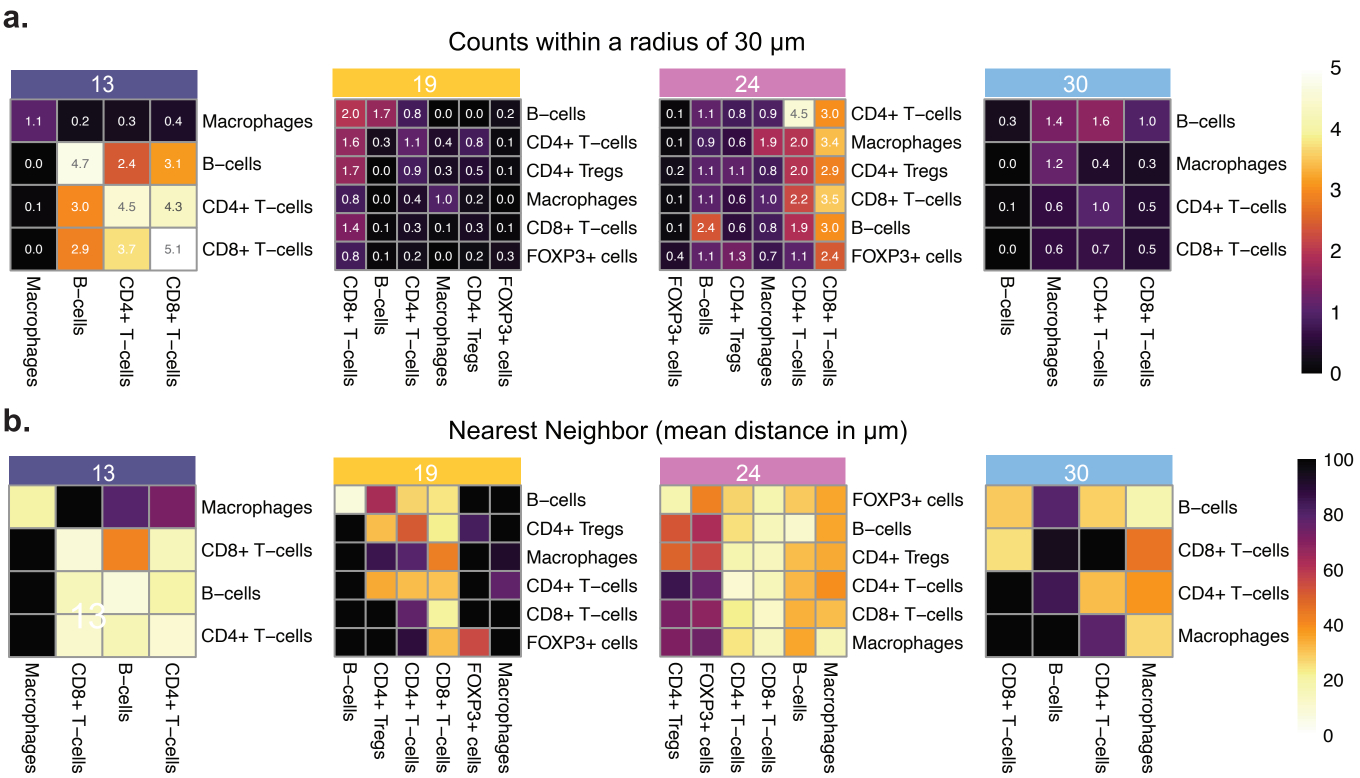

Supplementary Figure 6: Calculations of spatial colocalization metrics using the phenoptrReports package. a. Mean number of target cell type (x-axis) within 30µm of each reference cell type (y-axis), per patient, calculated by the phenoptrReports package for R[29]. **b.** Nearest neighbor distances (in micrometers) from each reference cell (y-axis) to each target cell type (x-axis), per patient. The distances have been reported as the mean of each tissue core's median distance.
