# Supplementary material for: Spatial Multiomics Reveals Intratumoral Immune Heterogeneity with Distinct Cytokine Networks in Lung Cancer Brain Metastases
Source: Cancer Res Commun. 2024 Nov 6;4(11):2888–902. doi: 10.1158/2767-9764.CRC-24-0201 (PMC11539001; doi:10.1158/2767-9764.CRC-24-0201)
Supplement: Supplementary Figure S8 — S8. Differentially Expressed Genes between TI and TC ROIs. [file crc-24-0201_supplementary_figure_s8_suppsf8.pdf]

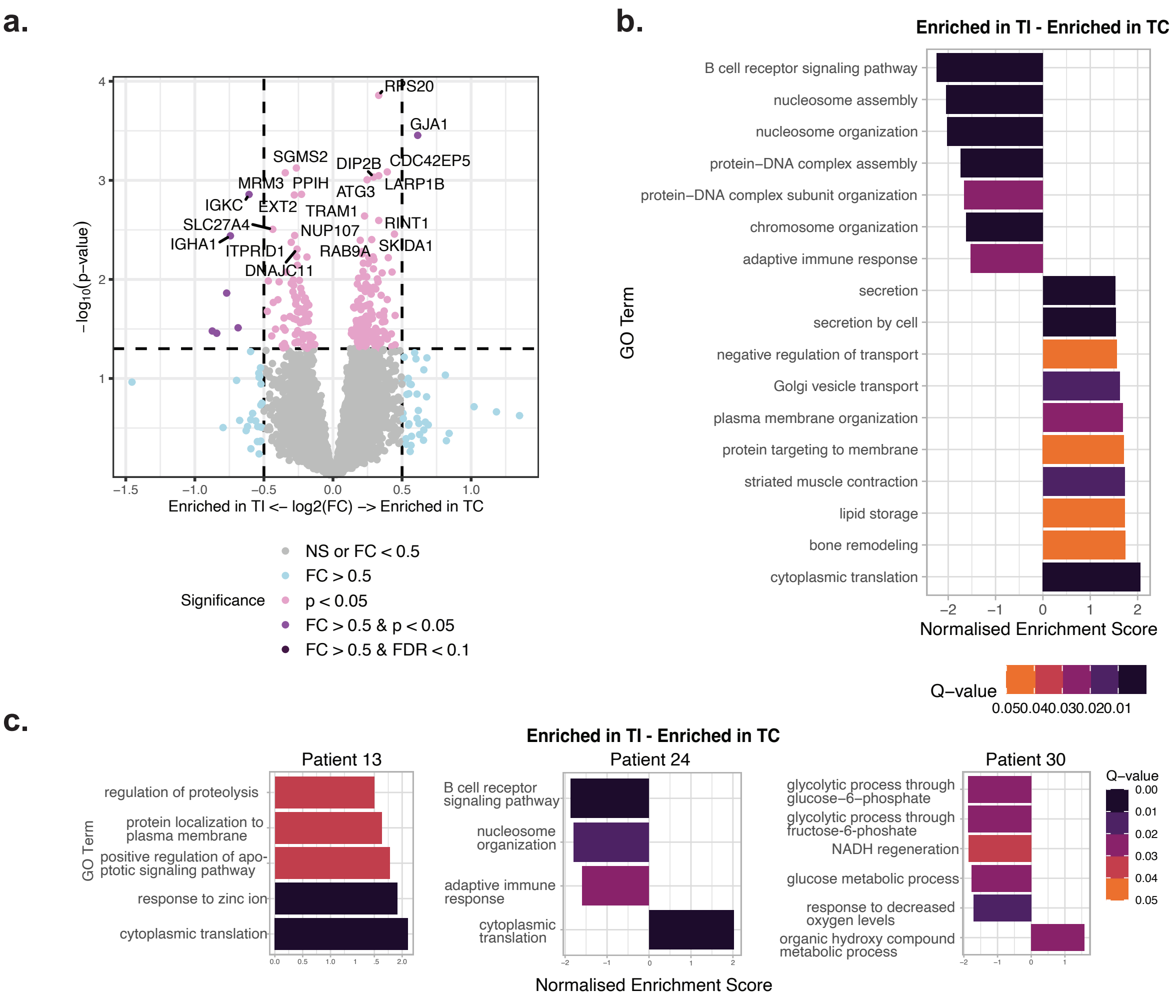

Supplementary Figure 8: Differentially Expressed Genes between TI and TC ROIs.
a. Volcano plot of differentially expressed genes between TI (left) and TC ROIs (right) for all patients. *p*-value cutoff was set to 0.05, whereas log2-fold-change cutoff was set to 0.50. **b.** Bar plot of top ontologies from the Gene Set Enrichment Analysis on the differentially expressed genes. *q*-value cutoff has been set to 0.05. **c.** Bar plots of top ontologies from the Gene Set Enrichment Analysis on the differentially expressed genes for patients 13, 24, and 30. The ontologies were chosen by picking the top five upregulated and downregulated ontologies, where they were present.
