# Supplementary material for: Spatial Multiomics Reveals Intratumoral Immune Heterogeneity with Distinct Cytokine Networks in Lung Cancer Brain Metastases
Source: Cancer Res Commun. 2024 Nov 6;4(11):2888–902. doi: 10.1158/2767-9764.CRC-24-0201 (PMC11539001; doi:10.1158/2767-9764.CRC-24-0201)
Supplement: Supplementary Figure S9 — S9. Immune-MTC cellular Networks in Lung Cancer Brain Metastasis. [file crc-24-0201_supplementary_figure_s9_suppsf9.pdf]

a.

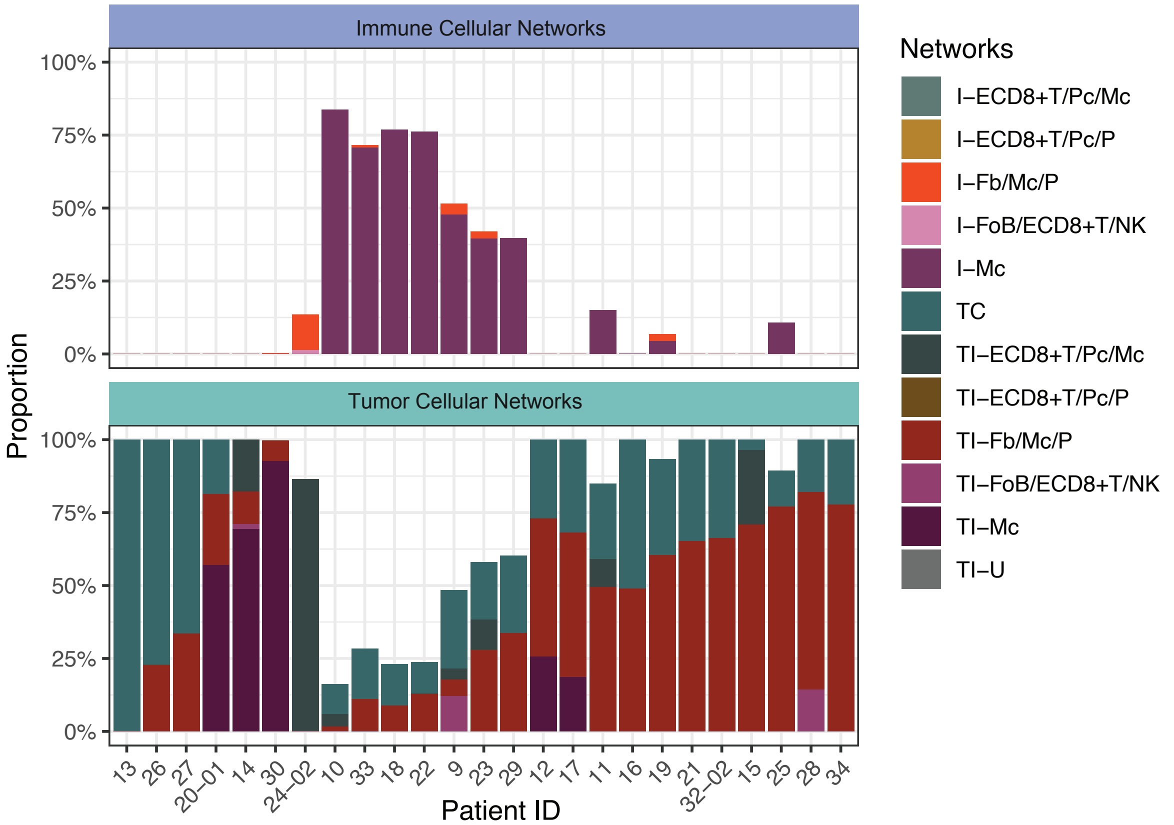

b.

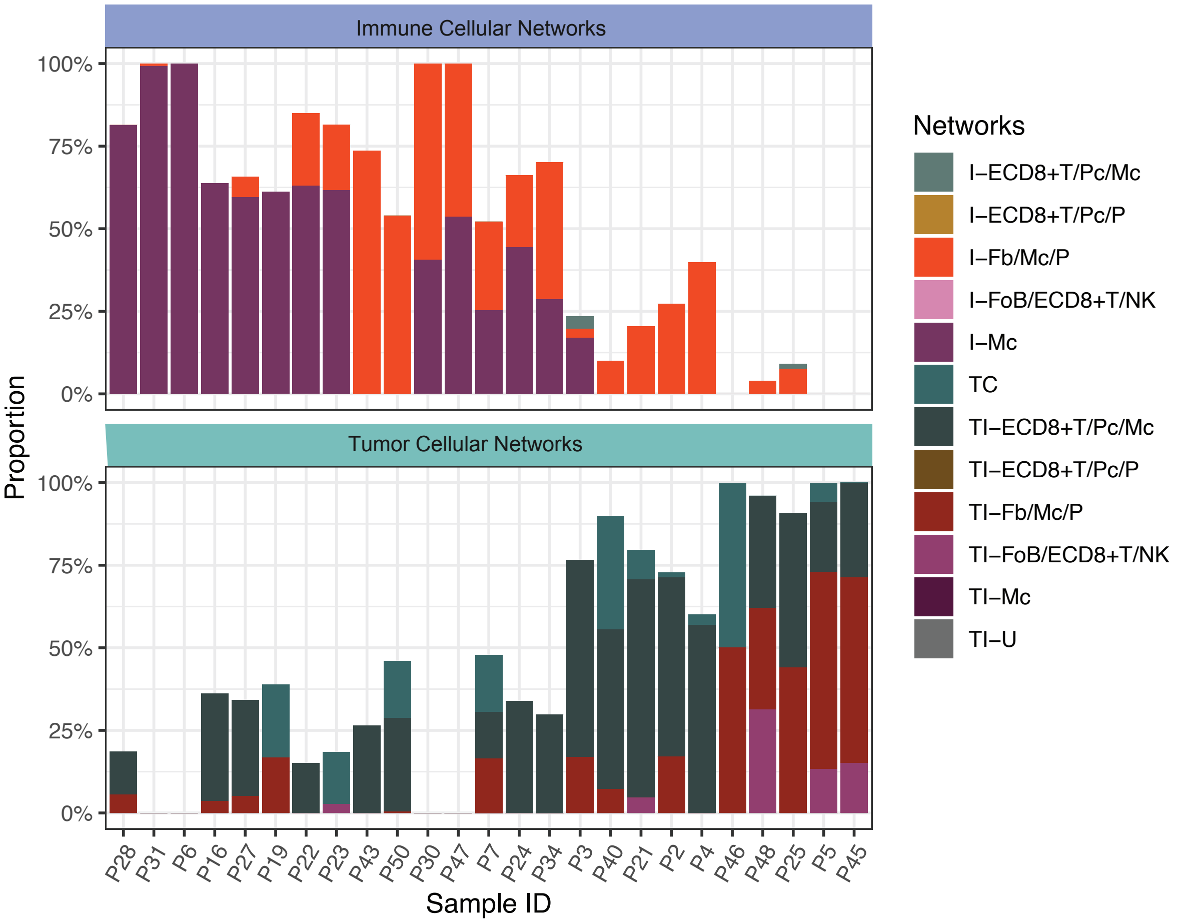

Supplementary Figure 9: Immune-MTC cellular Networks in Lung Cancer Brain Metastasis. **a.** Estimated cell proportions in our lung cancer brain metastasis cohort according to bulk RNA deconvolution with the GeoMx dataset as reference, showing data for all patients. **b.** Estimated cell proportions in the Rubio-Perez et al. dataset[31] according to bulk RNA deconvolution with the GeoMx dataset as reference, showing data for all patients. Patients have been clustered by their estimated proportions using hierarchical clustering and the “complete” agglomeration method from the ‘hclust’ package in R.
