# Supplementary material for: Spatial Multiomics Reveals Intratumoral Immune Heterogeneity with Distinct Cytokine Networks in Lung Cancer Brain Metastases
Source: Cancer Res Commun. 2024 Nov 6;4(11):2888–902. doi: 10.1158/2767-9764.CRC-24-0201 (PMC11539001; doi:10.1158/2767-9764.CRC-24-0201)
Supplement: Supplementary Figure S7 — S7. Differentially expressed (DE) genes for the immune infiltrate-associated TI ROIs. [file crc-24-0201_supplementary_figure_s7_suppsf7.pdf]

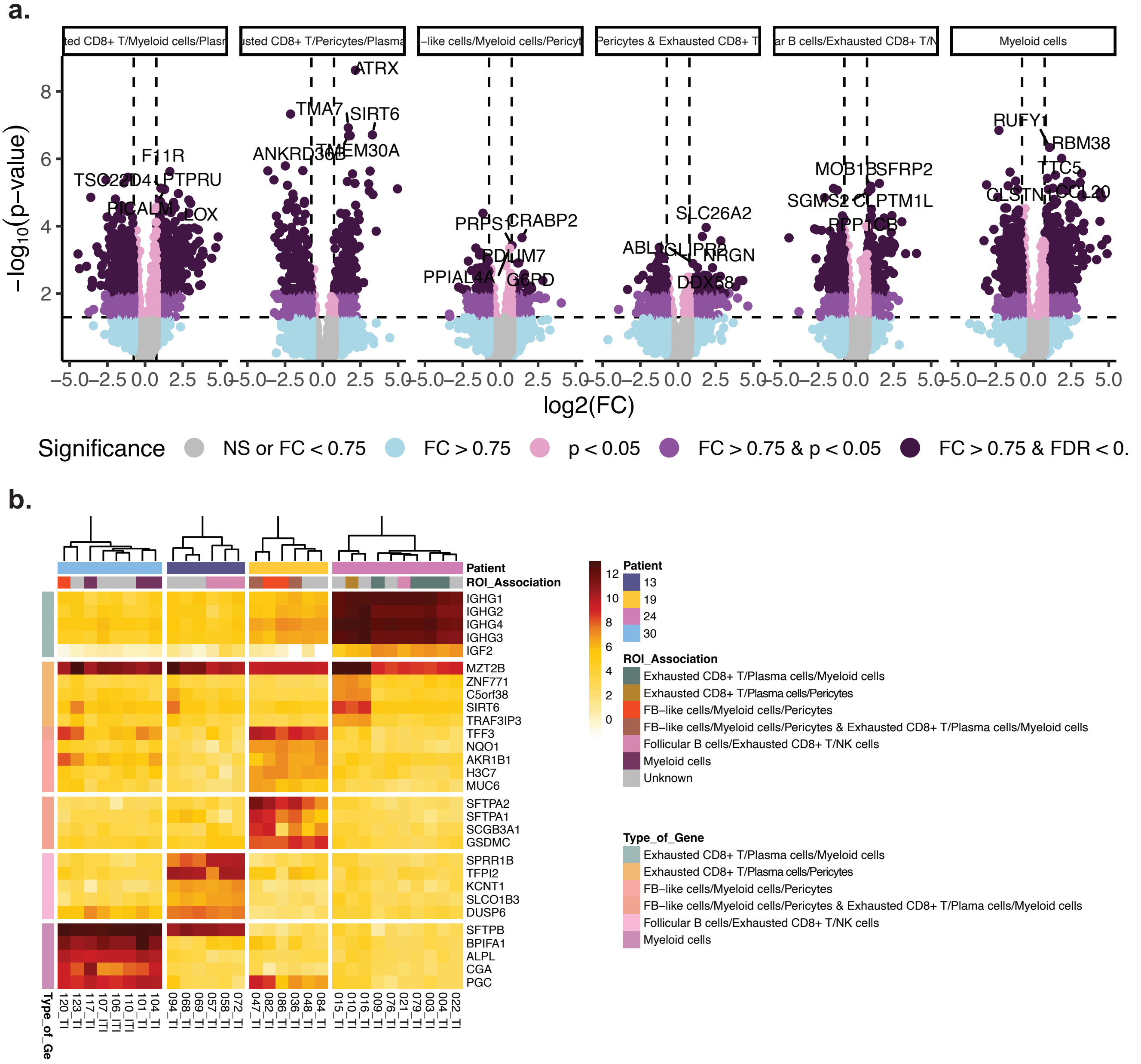

Supplementary Figure 7: Differentially expressed (DE) genes for the immune infiltrate-associated TI ROIs. a. Volcano plot of the DE genes, using 0.75 as log2-fold-change cutoff, 0.05 as *p*-value cutoff, and 0.10 as *q*-value cutoff. **b.** Gene expression of selected DE genes plotted across the TI segments.
